# Stabilizing hidden room-temperature ferroelectricity via a metastable atomic distortion pattern

**DOI:** 10.1038/s41467-020-18741-w

**Published:** 2020-10-02

**Authors:** Jeong Rae Kim, Jinhyuk Jang, Kyoung-June Go, Se Young Park, Chang Jae Roh, John Bonini, Jinkwon Kim, Han Gyeol Lee, Karin M. Rabe, Jong Seok Lee, Si-Young Choi, Tae Won Noh, Daesu Lee

**Affiliations:** 1 Center for Correlated Electron Systems, Institute for Basic Science (IBS), Seoul, 08826 Korea; 2 Department of Physics and Astronomy, Seoul National University, Seoul, 08826 Korea; 3 Department of Materials Science and Engineering, Pohang University of Science and Technology (POSTECH), Pohang, 37673 Korea; 4 Department of Physics, Soongsil University, Seoul, 07027 Korea; 5 Department of Physics and Photon Science, Gwangju Institute of Science and Technology (GIST), Gwangju, 61005 Korea; 6 Department of Physics and Astronomy, Rutgers University, Piscataway, NJ 08854-8019 USA; 7 Department of Physics, Pohang University of Science and Technology (POSTECH), Pohang, 37673 Korea; 8 Asia Pacific Center for Theoretical Physics, Pohang, 37673 Korea

**Keywords:** Ferroelectrics and multiferroics, Surfaces, interfaces and thin films

## Abstract

Nonequilibrium atomic structures can host exotic and technologically relevant properties in otherwise conventional materials. Oxygen octahedral rotation forms a fundamental atomic distortion in perovskite oxides, but only a few patterns are predominantly present at equilibrium. This has restricted the range of possible properties and functions of perovskite oxides, necessitating the utilization of nonequilibrium patterns of octahedral rotation. Here, we report that a designed metastable pattern of octahedral rotation leads to robust room-temperature ferroelectricity in CaTiO_3_, which is otherwise nonpolar down to 0 K. Guided by density-functional theory, we selectively stabilize the metastable pattern, distinct from the equilibrium pattern and cooperative with ferroelectricity, in heteroepitaxial films of CaTiO_3_. Atomic-scale imaging combined with deep neural network analysis confirms a close correlation between the metastable pattern and ferroelectricity. This work reveals a hidden but functional pattern of oxygen octahedral rotation and opens avenues for designing multifunctional materials.

## Abstract (web summary)

Previous studies are limited to modulate oxygen octahedral rotation angles within the same pattern, and engineering the pattern remains a challenge. Here, the authors demonstrate a designed nonequilibrium oxygen octahedral rotation pattern leads to room-temperature ferroelectricity in CaTiO_3_.

## Introduction

**Fig. 1:**
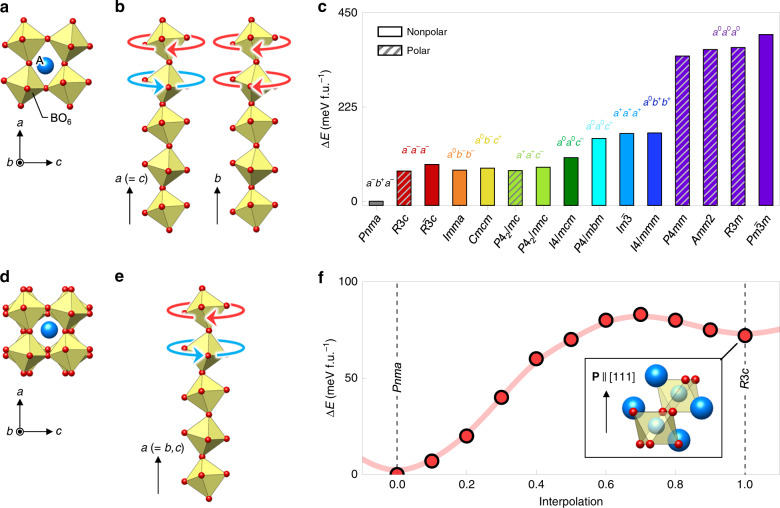
Hidden ferroelectricity of CaTiO_3_ in its metastable oxygen octahedral rotation (OOR) pattern. **a** Atomic structure of ABO_3_ perovskite with an *a*^−^*b*^+^*a*^–^ OOR pattern (*Pnma*). **b** *a*^−^*b*^+^*a*^−^ OOR pattern, showing out-of-phase rotation along the *a*- and *c*-axes and in-phase rotation along the *b-*axis. **c** Density-functional theory (DFT) calculation of the energy landscape for various OOR patterns of CaTiO_3_. The total energy for *Pnma* is set to zero. Five structures (*R*3*c*, *P*4_2_/*mc*, *P*4*mm*, *Amm*2, and *R*3*m*) are predicted to be polar. **d** Atomic structure of ABO_3_ perovskite with an *a*^−^*a*^−^*a*^−^ OOR pattern (*R*3*c* or $R\bar 3c$). **e** *a*^−^*a*^−^*a*^−^ OOR pattern, showing out-of-phase rotation along all the axes. **f** Energy barrier between the *Pnma* and metastable *R*3*c* phases of CaTiO_3_. We interpolate the transition between the *Pnma* and *R*3*c* structures by smoothly changing the OOR along the *b-*axis.

Despite their simple structure, ABO_3_ perovskites offer a wide range of functionalities including superconductivity, metal–insulator transition, ferroelectricity, and ferromagnetism. These functionalities largely originate from the rotation of corner-connected BO_6_ octahedra (Fig. 1a), which allows the ABO_3_ family to host a variety of cations. Furthermore, the angle and pattern of oxygen octahedral rotation (OOR)^1^ directly influence electrical conductivity^2^, magnetic superexchange interaction^3^, dielectric properties^4^, and so on. While this allows OOR to serve as a fundamental parameter for understanding functional perovskites, most perovskite bulks adopt the orthorhombic (*Pnma*) structure, corresponding to the *a*^–^*b*^+^*a*^–^ OOR pattern in Glazer notation (Fig. 1a, b)^5^. Such predominance of the *a*^–^*b*^+^*a*^–^ OOR pattern has prevented full exploitation of functional perovskites.

In particular, the most common *a*^–^*b*^+^*a*^–^ OOR pattern has been shown to compete with an important functional property, namely ferroelectricity^4^. As a result, most *Pnma* perovskites remain paraelectric, even down to 0 K, whereas their cubic phases could have instability for both ferroelectric and OOR distortions. This has motivated recent attempts to utilize nonequilibrium OOR, e.g., via artificial heteroepitaxy^6,7^. Despite extensive works^8–11^, however, there is still a lack of studies on engineering the pattern itself of OOR and then generating functionalities.

In this work, we designed a means to stabilize a nonequilibrium OOR pattern of CaTiO_3_ in oxide heterostructures. Unlike the original *a*^–^*b*^+^*a*^–^ pattern, the metastable OOR pattern appears to be compatible with ferroelectricity, leading to the emergence of room-temperature ferroelectricity. Moreover, we combined transmission electron microscopy techniques with deep neural network analysis and revealed the strong coupling between the metastable OOR pattern and ferroelectricity. Our work suggests that engineering the nonequilibrium OOR pattern unveils the hidden functionalities of materials.

## Results

### Theoretical search for nonequilibrium OOR patterns of CaTiO_3_

Taking CaTiO_3_—the first discovered perovskite compound—as a model system, we explore the feasibility of achieving ferroelectricity via its nonequilibrium OOR patterns (Supplementary Fig. S1). Bulk CaTiO_3_ has the *Pnma* space group symmetry and the *a*^–^*b*^+^*a*^–^ OOR pattern below 1512 K (ref. ^12^) and exhibits a stable nonpolar, paraelectric phase down to 0 K; bulk CaTiO_3_ exhibits antipolar Ca displacements. It is notable that CaTiO_3_ is an incipient ferroelectric material with a negative Curie–Weiss temperature^13–15^, so that it has potential to be engineered into a ferroelectric phase. Of the 23 possible OOR patterns, we focus on 10 patterns, since the other patterns rarely occur in a single ABO_3_ compound^5^. According to our density-functional theory (DFT) calculations (Fig. 1c), the nonpolar *Pnma* with the *a*^–^*b*^+^*a*^–^ OOR pattern has the lowest energy, consistent with the bulk crystal structure of CaTiO_3_. The highest energy is seen in the cubic $Pm\bar 3m$ without OOR (i.e., *a*^0^*a*^0^*a*^0^), which is highly unstable due to the small size of the A-site Ca ion^16^. The other eight OOR patterns are located in between and, to the best of our knowledge, have not been reported in bulk CaTiO_3_. Our theory predicts that among the eight OOR patterns, *a*^–^*a*^–^*a*^–^ and *a*^+^*a*^+^*c*^–^ allow for polar structures with the space groups *R*3*c* and *P*4_2_/*mc*, respectively. Given their moderate energy cost (as small as 100 meV f.u.^−1^), our DFT calculations indicate the possibility to thermodynamically stabilize the nonequilibrium *a*^–^*a*^–^*a*^–^ or *a*^+^*a*^+^*c*^–^ pattern and then achieve ferroelectricity in CaTiO_3_.

Here, we take *a*^–^*a*^–^*a*^–^ (Fig. 1d, e) as the target OOR pattern of CaTiO_3_ due to its experimental accessibility, which is discussed later. Our first-principles calculation finds no unstable phonon mode in the polar *R*3*c* with the *a*^–^*a*^–^*a*^–^ OOR pattern (Supplementary Fig. S3), indicating its metastability (Fig. 1f). The calculated polarization value, *P*, of the *R*3*c*-CaTiO_3_ is 44 μC cm^−2^, and the energy barrier, Δ*E*, for polarization switching is 16 meV f.u.^−1^. These values are comparable to those of archetypal ferroelectric perovskites^17^, such as BaTiO_3_ (*P* = 20 μC cm^−2^, Δ*E* = 11.6 meV f.u.^−1^) and PbTiO_3_ (*P* = 78 μC cm^−2^, Δ*E* = 32.6 meV f.u.^−1^), whose ferroelectric transition temperatures are 393 and 760 K, respectively. Therefore, artificial stabilization of the metastable *a*^–^*a*^–^*a*^–^ OOR pattern may result in robust room-temperature ferroelectricity—hitherto hidden at equilibrium—in CaTiO_3_.

### Heteroepitaxial stabilization of *R*3*c*-CaTiO_3_

**Fig. 2:**
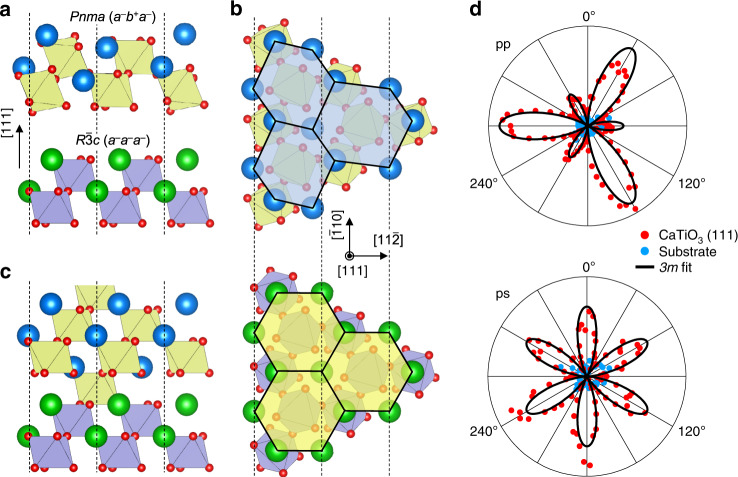
Heteroepitaxial stabilization of the *a*^–^*a*^–^*a*^–^ OOR pattern in CaTiO_3_. **a** Structural mismatch between the *a*^−^*b*^+^*a*^−^ and *a*^−^*a*^−^*a*^−^ OOR patterns in the (111) plane. **b** Disparate in-plane lattice symmetries of the *a*^−^*b*^+^*a*^−^ and *a*^−^*a*^−^*a*^−^ OOR patterns in the (111) plane. **c** Schematic diagram of OOR pattern engineering in the (111)-oriented heterointerface between *a*^−^*b*^+^*a*^−^ and *a*^−^*a*^−^*a*^−^ structures. **d** Optical second harmonic generation (SHG) signals from 2.6 nm-thick CaTiO_3_ (111) film (red) and LaAlO_3_ substrate (blue). “p” (“s”) indicates parallel (perpendicular) polarization of light with respect to the plane of incidence.

While the *R*3*c*-CaTiO_3_ is metastable in bulk, with a calculated energy barrier of 10 meV f.u.^−1^ (Fig. 1f), heteroepitaxial growth could make it more stable. Through simply modulating OOR angles^18^, it might be difficult to covert the OOR pattern itself from *a*^–^*b*^+^*a*^–^ (i.e., corresponding to *Pnma*) to *a*^–^*a*^–^*a*^–^ (i.e., corresponding to *R*3*c*). To overcome this, we constrain CaTiO_3_ to the pseudo-cubic (111)_pc_ plane of the LaAlO_3_ substrate, which has the *a*^–^*a*^–^*a*^–^ OOR pattern (note that we use a pseudo-cubic unit cell throughout and omit the pc subscript hereafter). Figure 2a, b emphasize that on the (111) plane, *a*^–^*b*^+^*a*^–^ and *a*^–^*a*^–^*a*^–^ OORs lead to disparate lattice symmetries; among the ten possible OOR patterns, only the *a*^–^*a*^–^*a*^–^ pattern allows for a regular hexagon network of A-site ions. Furthermore, the (111) interface maximizes the octahedral connectivity through three metal-oxygen-metal bonding (Supplementary Fig. S5), so that the OOR pattern of CaTiO_3_ could more likely follow that of LaAlO_3_. Accordingly, coherent, epitaxial growth of CaTiO_3_ films on an LaAlO_3_ (111) substrate could effectively stabilize the *a*^–^*a*^–^*a*^–^ OOR pattern (Fig. 2c) in CaTiO_3_.

We fabricate high-quality CaTiO_3_ films on an LaAlO_3_ (111) substrate using a pulsed laser deposition (PLD) technique (see “Methods”). As a nonequilibrium growth method^19^, PLD could assist in achieving the metastable *a*^–^*a*^–^*a*^–^ OOR pattern in CaTiO_3_ films. Atomic force microscopy (Supplementary Fig. S8b) and X-ray diffraction (XRD) (Supplementary Fig. S8c) confirm that the films have smooth surfaces and high crystallinity. Previous works have reported experimental difficulties in achieving sharp, (111)-oriented heterointerfaces of perovskite oxides due to their high surface polarity^20^. In this work, we utilize in situ annealing of an LaAlO_3_ substrate in ultra-high vacuum to achieve an atomically sharp CaTiO_3_/LaAlO_3_ (111) interface (“Methods” and Supplementary Fig. S9)^21^. Given this sharp interface, the OOR pattern in CaTiO_3_ (111) films could follow that (i.e., the *a*^–^*a*^–^*a*^–^ pattern) of the underlying LaAlO_3_ substrate.

### Optical characterization of polar symmetry in CaTiO_3_ (111) films

Using optical second harmonic generation (SHG) polarimetry, we identify the macroscopic polar point-group symmetry of CaTiO_3_ (111) films. Figure 2d shows a clear SHG signal in CaTiO_3_ (111) films, which best fits with the polar point group of 3*m*. This is consistent with our prediction that the CaTiO_3_/LaAlO_3_ (111) heteroepitaxy could stabilize the polar *R*3*c* phase with an *a*^–^*a*^–^*a*^–^ OOR pattern in CaTiO_3_, belonging to the polar point group of 3*m*. Furthermore, the SHG studies show a typical thickness dependence of ultrathin ferroelectric films. While we observe a persistent SHG signal in films thicker than 2.6 nm (i.e., 12 unit cells), the SHG signal disappears in films thinner than 1.3 nm (6 unit cells; Supplementary Fig. S11). This size effect is an intrinsic characteristic in ultrathin ferroelectrics^22^, wherein ferroelectricity disappears at a finite critical thickness, e.g., due to depolarization field. Therefore, the observed SHG signal should arise solely from the bulk of CaTiO_3_ (111) films, rather than from their surfaces and interfaces.

### Atomic-scale imaging of OOR patterns and polarization

**Fig. 3:**
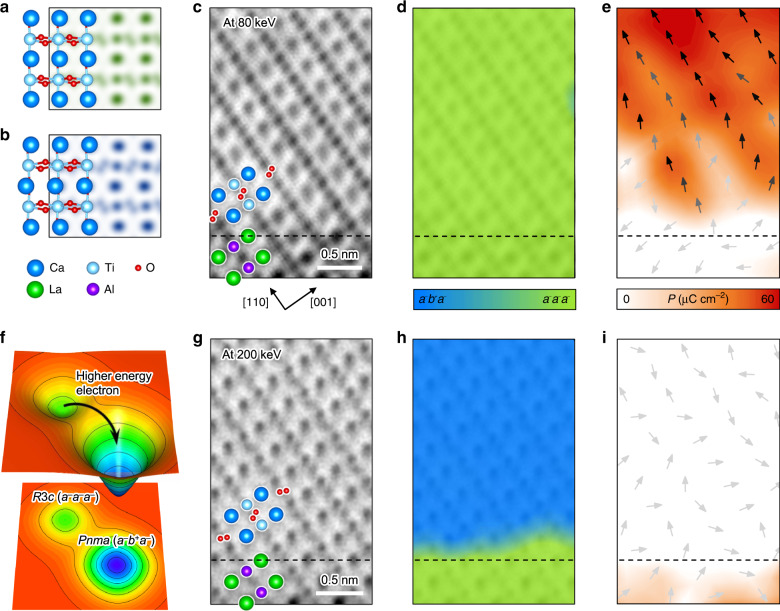
Atomic-scale analysis of the OOR pattern and polarity in CaTiO_3_ films. Simulated annular bright-field scanning transmission electron microscopy (ABF-STEM) images along the zone axis of [$1\bar 10$] for *a*^−^*a*^−^*a*^−^ (**a**) and [101] for *a*^−^*b*^+^*a*^−^ (**b**) OOR patterns. **c**–**i** (**c**) and (**g**) show ABF-STEM images of 2.6 nm-thick CaTiO_3_ (111) film along the zone axis of [$1\bar 10$], measured using 80-keV (**c**) and 200-keV (**g**) electron kinetic energy. **d**, **h** display the maps of OOR patterns, identified by deep neural network analysis, in the same regions as in (**c**) and (**g**), respectively. Color indicates the probability of each OOR pattern. **e**, **i** present polarization vectors for each unit cell of the same regions as in (**c**) and (**g**), respectively. Arrows denote the polarization direction; the stronger the polarization, the darker the arrow color. Strength of polarization is also expressed as a color map, ranging from white (weak) to red (strong). **f** illustrates a schematic free energy landscape, showing the relaxation of *R*3*c* state (*a*^−^*a*^−^*a*^−^) into *Pnma* state (*a*^−^*b*^+^*a*^−^).

Scanning transmission electron microscopy (STEM) can provide atomic-scale information on the OOR pattern, as well as ferroelectricity, of the films. In particular, annular bright-field STEM (ABF-STEM) images are sensitive to light atoms (e.g., oxygen)^23^, making them an effective tool for visualizing OOR patterns. Because OOR generally causes elongation of oxygen peaks in ABF-STEM images, qualitative analysis of such elongated oxygen peaks can provide the means to identify the OOR pattern in the films^24^. Indeed, our ABF-STEM simulations, based on the calculated atomic structures in Fig. 1c, indicate that each OOR pattern has its unique oxygen peak feature in the [1$\bar 1$0] projection (Supplementary Fig. S12): the *a*^–^*a*^–^*a*^–^ OOR pattern has the elongated oxygen peaks aligned in one crystalline direction (Fig. 3a) and the *a*^–^*b*^+^*a*^–^ pattern has the elongated oxygen peaks arranged in a zigzag-like pattern (Fig. 3b). Importantly, this unique feature of the oxygen peak shapes allows the OOR pattern of the CaTiO_3_ films to be identified.

Furthermore, we employ a deep neural network approach to identify the OOR patterns from the measured ABF-STEM images (see “Methods”)^25,26^. Identification of OOR patterns could be ambiguous, e.g., due to nontrivial experimental noise, difficulty in the quantification of shape information, or infinitesimal differences among OOR shapes. The deep neural network approach, however, can help us to identify OOR patterns beyond human cognition. Figure 3c displays the measured ABF-STEM image of CaTiO_3_ (111) films with a zone axis of [1$\bar 1$0], exhibiting oxygen peaks elongated along the same direction. This is consistent with the simulated ABF-STEM image for the *a*^–^*a*^–^*a*^–^ OOR pattern (Fig. 3a). Our deep neural network analysis indeed identifies the *a*^–^*a*^–^*a*^–^ OOR pattern from the measured ABF-STEM image of CaTiO_3_ (111) films (Fig. 3d). The ABF-STEM image also confirms that such an *a*^–^*a*^–^*a*^–^ OOR pattern coexists with electric polarization in CaTiO_3_ (111) films (Fig. 3e), which is in good agreement with our theoretical prediction and SHG results. [Fig. 3e shows rather suppressed electric polarization near the bottom interface, which might originate from interface dipoles (Supplementary Fig. S6).] In stark contrast, CaTiO_3_ (001) films, which differ from CaTiO_3_ (111) films only in their crystalline orientation, show the *a*^–^*b*^+^*a*^–^ OOR pattern and no electric polarization (Supplementary Fig. S13i–l). This is consistent with the nonpolar *Pnma* phase of bulk CaTiO_3_ (Fig. 3b). These results thus firmly validate our heteroepitaxial design principle for stabilizing the nonequilibrium OOR pattern and achieving polar CaTiO_3_ at room temperature.

### Demonstration of switchable polarization

**Fig. 4:**
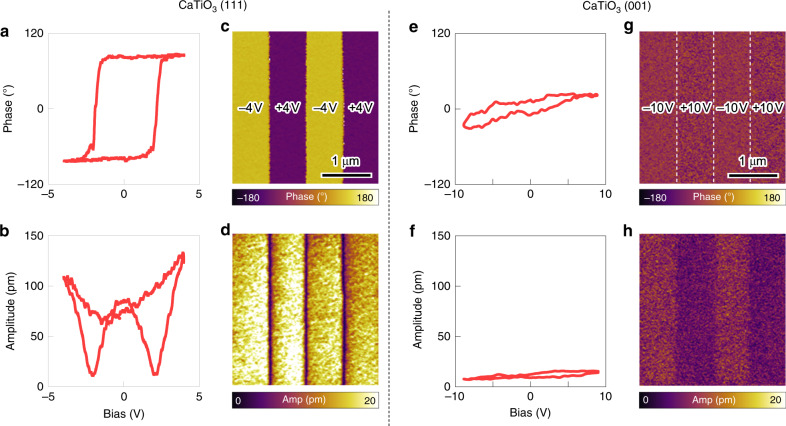
Switchable and stable polarization in CaTiO_3_ (111) films. Switching behavior of the piezoelectric phase (**a**) and amplitude (**b**) in 2.6 nm-thick CaTiO_3_ (111) film. Bipolar domain patterns, written on the same CaTiO_3_ (111) film. Piezoelectric phase (**c**) and amplitude (**d**) show two stable ferroelectric domains. Nonferroelectric behavior of the piezoelectric phase (**e**) and amplitude (**f**) in 3.8 nm-thick CaTiO_3_ (001) film. The absence of domain patterns in the same CaTiO_3_ (001) film. Piezoelectric phase (**g**) and amplitude (**h**) show the absence of ferroelectric domains.

Then, using piezoresponse force microscopy (PFM), we examine whether the electric polarization is switchable and stable—the signature of ferroelectricity—at room temperature. To apply an electric field, we prepare CaTiO_3_ films on an LaAlO_3_ substrate buffered with LaNiO_3_ films as the bottom electrode. Given that LaNiO_3_ has the $R\bar 3c$ structure with an *a*^–^*a*^–^*a*^–^ OOR pattern, the same heteroepitaxy control of the OOR patterns in CaTiO_3_ (Fig. 2c) would work. Our PFM measurement reveals clear hysteretic behavior (Fig. 4a, b), with a phase difference of around 180° in CaTiO_3_ (111) films, consistent with the typical ferroelectric responses under PFM. In contrast, CaTiO_3_ (001) films show almost no change in the PFM phase, and a negligible PFM amplitude (Fig. 4e, f); accordingly, we exclude extrinsic electrochemical effects^27^ as the origin of the PFM response in (111) films. Figure 4 verifies that the [111]-directed film growth is essential in stabilizing the ferroelectric state, as it not only guarantees the unique geometric constraint (Fig. 2), but also maximizes octahedral connectivity (Supplementary Fig. S5). Bipolar domain patterns (Fig. 4c, d) were writable and stable on CaTiO_3_ (111) films. The pristine domain structure of CaTiO_3_ (111) films was difficult to define (Supplementary Fig. S14); however, it may be attributable to the ferroelectric domain structure (Supplementary Fig. S18), which can assume a mono-domain configuration upon electrical poling (Fig. 4c, d).

We examined the stability of the written domains by monitoring the PFM signal as a function of time (Supplementary Fig. S15). The PFM signal gradually decays, following a power-law with a decay exponent of 0.145. Although comparable decay exponents have been reported in the relaxor ferroelectrics^28^, a similar relaxation has also been observed in prototypical ferroelectric films at the ultrathin limit^29^. Based on our comprehensive DFT and STEM studies, we attribute the relaxation behavior of the ferroelectric *R3c*-CaTiO_3_ to the huge depolarization field in ultrathin ferroelectric films. Taken together, our theoretical and experimental results consistently demonstrate that artificial stabilization of the nonequilibrium OOR pattern leads to robust room-temperature ferroelectricity in otherwise nonpolar CaTiO_3_ films.

## Discussion

Our results highlight that we achieve both selective stabilization and atomic-scale imaging of the ferroelectrically active, nonequilibrium *a*^–^*a*^–^*a*^–^ OOR pattern. Importantly, because nonequilibrium states inherently involve complex structural instabilities, it has been difficult to selectively stabilize them and resolve their atomic structures^30^. However, by using the moderate conditions in the STEM experiments (e.g., electron kinetic energy of 80 keV), we successfully resolve the artificially stabilized *a*^–^*a*^–^*a*^–^ OOR pattern at the atomic scale, without structural relaxation to the ground state (*a*^–^*b*^+^*a*^–^ pattern). This implies that STEM imaging with higher electron energy would cause greater perturbation of the system, possibly resulting in structural relaxation from the *a*^–^*a*^–^*a*^–^ to the *a*^–^*b*^+^*a*^–^ OOR pattern (Fig. 3f). Indeed, when imaged with a higher electron kinetic energy (200 keV), the atomic structure relaxes to that of the *a*^–^*b*^+^*a*^–^ OOR pattern, identified via deep neural network analysis (Fig. 3g, h). Importantly, this relaxation of the OOR pattern coincides with the disappearance of electric polarization (Fig. 3i). Furthermore, even after the OOR pattern is relaxed to *a*^–^*b*^+^*a*^–^, CaTiO_3_ still remains fully strained to the underlying LaAlO_3_ substrate (Supplementary Fig. S16). This emphasizes that the epitaxial strain itself cannot stabilize the *a*^–^*a*^–^*a*^–^ OOR pattern and resulting ferroelectricity in CaTiO_3_/LaAlO_3_ heterostructures, consistent with our DFT calculations (Supplementary Fig. S4). Thus, these results not only confirm a close correlation between the metastable *a*^–^*a*^–^*a*^–^ OOR pattern and ferroelectricity, but also exclude the epitaxial strain as a primary origin for the ferroelectricity in CaTiO_3_ (111) films.

Interest in understanding and utilizing metastable or hidden states, which would allow for exotic phenomena and functions, has been growing^30–34^. While optical pumping has been mainly used to explore nonequilibrium transient states^33^, we stabilize a nonequilibrium OOR pattern and then achieve robust ferroelectricity. Our approach to utilize metastable OOR patterns for functional perovskite oxides is distinct from conventional chemical substitution^35^ or strain^36^/dimensional^29^ engineering. Moreover, our theory suggests that the metastable OOR pattern may facilitate the development of multifunctionalities (Supplementary Fig. S17): our results indicate that in the metastable *a*^–^*a*^–^*a*^–^ OOR pattern, an increase in rotation angle could enhance the electric polarization, in stark contrast to the conventional *a*^–^*b*^+^*a*^–^ pattern (which is competitive with ferroelectricity). Such cooperation between the OOR and polar distortion could constitute a generic principle for polarizing various material systems, including not only dielectrics but also conductors. Thus, OOR pattern engineering is expected to be widely used to design multifunctional perovskite oxides, in which the broken inversion symmetry is combined with electron conduction, magnetism, and topological phases.

## Methods

### Thin film growth

Commercially available LaAlO_3_ single crystal substrates (Crystec GmbH, Germany) were used. Prior to the growth, LaAlO_3_ (111) and (001) substrates were dipped in deionized water and sonicated for 2 h. Then, the substrates were annealed in the growth chamber in situ. The annealing temperature, background gas pressure, and annealing time were 1000 °C, <1.0 × 10^–7^ mTorr, and 1 h, respectively. Using this procedure for preparing substrates, we realized clean (111)-oriented heterointerfaces despite the high surface polarity (Fig. S9). We observed no structural defects or interfacial disorder over a large area (Fig. S9a, d)^20^. The small-scale images (Fig. S9b, c, e, f) confirm that the interfacial atomic structure is abrupt and epitaxial.

CaTiO_3_ and LaNiO_3_ thin films were grown using PLD. For the growth of thin films, the substrate temperature and background oxygen partial pressure were kept at 600 °C and 10 mTorr, respectively. Polycrystalline Ca_1.1_TiO_3.1_ and LaNiO_3_ targets were ablated with a KrF excimer laser (LPXpro, Coherent, USA). Off-stoichiometry of the cation in oxide dielectrics can produce defect dipoles and ferroelectricity^29^, which are undesirable in this work. To exclude such extrinsic properties, we optimized the growth of cation-stoichiometric CaTiO_3_ thin films. In the growth of CaTiO_3_ thin films, the laser energy density played a critical role in determining film quality. Figure S7a–f shows the surface morphology and reflection high energy electron diffraction (RHEED) patterns of CaTiO_3_/LaAlO_3_ (001) thin films grown with the laser energy density of 0.5, 1.0, and 1.5 J cm^–2^. We noticed that a smooth surface and spotty RHEED pattern are only achieved with an optimal laser energy density of 1.0 J cm^–2^. When the value is too low, cracked surfaces can occur. On the other hand, when it is too high, rough surfaces and extra peaks in RHEED patterns have been observed. This feature is consistent with the off-stoichiometric CaTiO_3_ thin film, reported previously^37,38^. In addition, the CaTiO_3_ thin film grown on a (LaAlO_3_)_0.3_–(Sr_2_AlTaO_6_)_0.7_ (LSAT) (001) substrate with optimal laser energy density has the same *c*-axis lattice parameter as films grown by molecular beam epitaxy (Fig. S7g). We examined the possible oxygen vacancy formation in our optimal CaTiO_3_ film by postannealing in oxygen. The postannealing temperature, background gas pressure, and annealing time were 500 °C, 760 Torr of pure oxygen, and 2 h, respectively. This postannealing process did not change the lattice parameter of our optimal CaTiO_3_, implying that the sample is already well-oxidized. Thus, our CaTiO_3_ thin films grown with optimal laser energy density should be reasonably stoichiometric in terms of cation and oxygen. Throughout this work, we used the optimal laser energy density for growing CaTiO_3_ thin films.

We fabricated CaTiO_3_/LaAlO_3_ (111) heterostructures using the same growth condition as for CaTiO_3_/LaAlO_3_ (001). The in situ RHEED intensity monitoring shown in Fig. S8a indicates layer-by-layer growth of CaTiO_3_ on the LaAlO_3_ (111) substrate. The grown films had smooth surfaces with step and terrace structures (Fig. S8b).

### Characterization of thin films

XRD (AXS D8 with a Vantec line-detector, Bruker, USA) was used for structural analysis of thin films. All films were epitaxially grown on the LaAlO_3_ (111) substrates and clear Kiessig fringes are seen (Fig. S8c). For ultrathin films with a thickness of several nanometers, we used Huber six-circle diffractometers at Sector 3A of the Pohang Light Source.

Switchable ferroelectric polarization was confirmed using scanning probe microscopy (Cypher, Asylum Research, UK) with conductive probes (PPP-EFM, Nanosensors). Dual AC resonance tracking PFM was used to read/write bipolar domain patterns and obtain *d*_33_ values as a function of the applied voltage. To exclude the contribution of electrostatic force to PFM signals, PFM was performed at higher-harmonic resonance frequency near 900 kHz.

Switching spectroscopy PFM measurements were also carried out on CaTiO_3_/LaNiO_3_/LaAlO_3_ (001) films as well. Bias-dependent PFM phase and amplitude measurements show no hysteresis loop up to the maximum bias voltage of 10 V (Fig. 4e, f). We attempted to write bipolar domains as in the case of (111)-oriented CaTiO_3_ (Fig. 4g, h). However, the phase difference between the two domains was small and the amplitude of the two domains was not equivalent; this is characteristic of electrostatic charge injection rather than ferroelectricity, which further corroborates the absence of ferroelectricity in (001)-oriented CaTiO_3_ films.

### Density-functional theory calculation

We performed first-principles DFT calculations within the local density approximation^39,40^ using the Vienna ab initio simulation package (VASP)^41,42^. The projector augmented wave method^43^ was used with an energy cut-off of 500 eV. The Brillouin zone was sampled with an 8 × 8 × 8 *k*-point grid for the 5-atom unit cell of rhombohedral CaTiO_3_ and a 4 × 4 × 4 *k*-point grid is used for a 2 × 2 × 2 supercell with 40 atoms to accommodate the relevant octahedral rotations and polar distortions. Convergence was reached if the consecutive energy difference is less than 10^–6^ eV. The structural relaxation was conducted with a force threshold of 0.001 eV Å^–1^. The polarization was calculated using the Berry-phase method^44^ as implemented in VASP. The phonon dispersion was calculated using the density-functional perturbation theory implemented in the VASP and phonopy^45^ with an increased energy cut-off (600 eV) and convergence threshold (10^–8^ eV).

Locally stable polar structures for each of ten rotation patterns are obtained by evaluating the phonon dispersion of a 2 × 2 × 2 supercell (Fig. 1c). The ten rotation patterns were chosen based on reported rotation patterns of single-phase perovskite oxides^5^. The systematic search procedure to obtain locally stable polar structures is presented in Fig. S1.

With the *a*^–^*a*^–^*a*^–^ OOR pattern, CaTiO_3_ can either be polar *R*3*c* or nonpolar $R\bar 3c$ (Fig. S2). Based on the DFT calculation of LaAlO_3_, *Pnma*-CaTiO_3_, $R\bar 3c$-CaTiO_3_, and *R*3*c*-CaTiO_3_, we estimated the lattice mismatch between CaTiO_3_ and LaAlO_3_. The results are summarized in Table S1. Overall, CaTiO_3_ films are in small compressive strain on LaAlO_3_ substrate.

We calculated phonon dispersion of CaTiO_3_ with $R\bar 3c$ and *R*3*c* structures to check the ferroelectricity of CaTiO_3_ in the given OOR pattern of *a*^–^*a*^–^*a*^–^ (Fig. S3). $R\bar 3c$-CaTiO_3_ has imaginary polar modes at the *Г* point (Fig. S3a), so it must relax to other stable or locally stable structures. After following the flowchart in Fig. S1, $R\bar 3c$-CaTiO_3_ was relaxed to the *R*3*c*-CaTiO_3_ rather than the ground state (*Pnma*-CaTiO_3_). The *R*3*c*-CaTiO_3_ did not show any unstable phonon modes (Fig. S3b). This local stability of *R*3*c*-CaTiO_3_ implies that stabilization of the *a*^–^*a*^–^*a*^–^ OOR pattern can realize the ferroelectricity in CaTiO_3_.

To understand the role of epitaxial strain in the CaTiO_3_/LaAlO_3_ (111) heterostructures, we calculated the energy of *Pnma* and *R*3*c* structures under biaxial strain (Fig. S4a). Under compressive or tensile strain up to 2%, *Pnma* always has lower energy than *R*3*c*. Furthermore, strain-dependent phonon dispersion calculations on the *Pnma* structure indicated the robust stability of the *Pnma* structure under biaxial strain (Fig. S4b–d). This excludes a significant role of the epitaxial strain in stabilizing *R*3*c*-CaTiO_3_ in CaTiO_3_/LaAlO_3_ (111) heterostructures. Instead, the surface symmetry of LaAlO_3_ (111) substrates should play the dominant role in stabilizing *R*3*c*-CaTiO_3_.

We investigated the relation between OOR and ferroelectricity in *R*3*c*-CaTiO_3_. Here, the OOR angle is defined by the amount of rotation perpendicular to the (111)-axis (Fig. S17a). The calculated strain- and OOR angle-dependent polarization is plotted in Fig. S17b. Overall, the polarization increases with the OOR angle, implying a cooperative coupling between OOR and ferroelectricity in *R*3*c*-CaTiO_3_^46^. The cooperative coupling was enhanced under compressive strain, which was our experimental condition.

To explain the suppressed electric polarization of CaTiO_3_ in the CaTiO_3_/LaAlO_3_ (111) interfacial region (Fig. 3e), we carried out DFT calculation of realistic heterostructure composed of *R*3*c*-CaTiO_3_ and LaNiO_3_. In the case of CaTiO_3_/LaAlO_3_ interface, we found that electrons or holes are doped in CaTiO_3_ depending on interfacial termination configurations^47^, which make our calculation complicated. Replacing LaAlO_3_ with LaNiO_3_ does not dope any charge carriers into the CaTiO_3_ and will not affect our arguments on interface dipole. Figure S6 shows the DFT calculation of [(LaNiO_3_)_12_/(CaTiO_3_)_12_] superlattices with Ni/CaO_3_ and LaO_3_/Ti interface terminations. Independent of interface termination, interface dipoles are always pointing from CaTiO_3_ to LaNiO_3_. When intrinsic polarization of CaTiO_3_ is pointing the opposite direction (11th or 12th layer in Fig. S6c, d), polarization is suppressed in the interfacial region and it is consistent with our observation.

### Second harmonic generation experiment

The symmetry of CaTiO_3_ thin films grown on (001) and (111) LaAlO_3_ substrate was characterized by SHG. Figure S10 displays the experimental set-up for the SHG. The 800-nm femtosecond wave, with an 80-MHz repetition rate and 30-fs duration, was irradiated on the thin films with an incidence angle of 45° as a fundamental wave (Vitara-T, Coherent). We focused the beam spot size into 1–30 μm. The polarization states of a fundamental and generated second harmonic wave were controlled to be p- or s-polarization by a half-wave plate and polarizer, respectively. To avoid the detection of the fundamental wave, we isolated the second harmonic wave using the short pass and bandpass filters. We monitored the intensity of the second harmonic response using a photomultiplier tube at the end of the optical path.

The allowed nonlinear susceptibility (*χ*) components of point group 3*m* are *χ*_xxx_ = −*χ*_xyy_ = −*χ*_yxy_ = −*χ*_yyx_, *χ*_zxx_ = *χ*_zyy_, *χ*_xxz_ = *χ*_yyz_, *χ*_xzx_ = *χ*_yzy_, and *χ*_zzz_. We calculated and fitted the polarization-dependent SHG intensity from CaTiO_3_ thin films, using the formulas below:

1 $$\left\{ \begin{array}{l} I_{{\mathrm{pp}}}(2\omega ) = \left[ {(\chi _{zyy} - \chi _{yzy} - \chi _{yyz} + \chi _{zzz}) + \chi _{xxx}\sin (3\varphi )} \right]^2\\ I_{{\mathrm{sp}}}(2\omega ) = 4\left[ {\chi _{zyy} - \chi _{xxx}\sin (3\varphi )} \right]^2\hfill\\ I_{{\mathrm{ps}}}(2\omega ) = \left[ {\chi _{xxx}\cos (3\varphi )} \right]^2\hfill\\ I_{{\mathrm{ss}}}(2\omega ) = 4\left[ {\chi _{xxx}\cos (3\varphi )} \right]^2\hfill\end{array} \right..$$

We carried out SHG experiments on 2.6 and 1.3 nm-thick CaTiO_3_/LaAlO_3_ (111) thin films (Figs. 2d and S11). In the case of 2.6 nm-thick films, the observed SHG was well fitted with a point group 3*m*. On the other hand, negligible SHG was detected in the 1.3 nm-thick films. Considering that most ferroelectrics lose their ferroelectricity below a finite thickness, it is expected that CaTiO_3_ grown on LaAlO_3_ (111) has a critical thickness for ferroelectricity between 1.3 and 2.6 nm. The critical thickness confirms that our observation is from neither the CaTiO_3_ surface nor the CaTiO_3_/LaAlO_3_ interface. In addition, the possibility of defect-induced ferroelectricity, which is enhanced with reduced film thickness^29^, is also ruled out.

### Scanning transmission electron microscopy

The CaTiO_3_/LaAlO_3_ (111) sample has a metastable *R*3*c* phase with an *a*^–^*a*^–^*a*^–^ OOR pattern which can be relaxed to *a Pnma* phase with an *a*^–^*b*^+^*a*^–^ OOR pattern; thus, we used mild sample preparation and STEM imaging conditions for the CaTiO_3_/LaAlO_3_ (111) sample. Cross-sectional specimens oriented along the [$1\bar 10$] direction of both samples were prepared by conventional mechanical flat polishing with less than 10 μm thickness. Mechanically polished samples were further milled using a 3 keV Ar ion beam for CaTiO_3_/LaAlO_3_ (001) and 2 keV for CaTiO_3_/LaAlO_3_ (111) and using a 0.1 keV Ar ion beam for removing surface damage from both samples (PIPS II, Gatan, USA). ABF-STEM images for light elements, i.e., oxygen, were acquired using STEM (ARM 200F, JEOL, Japan) with a spherical aberration corrector (ASCOR, CEOS GmbH, Germany) at Materials Imaging & Analysis Center of POSTECH in South Korea. Additionally, to minimize the electron beam-induced phase transition of the metastable CaTiO_3_/LaAlO_3_ (111) sample, imaging was also conducted at an 80-kV accelerating voltage with a convergence angle of 27 mrad.

The standard position of each unit cell was determined from the Ti- or Al-atomic column, and the positive charge position was defined by the six-neighboring Ca- or La-atomic column (because the Ca-atomic column off-centering is larger than that for Ti). The negative charge positions are disregarded under the assumption that the O and Ca ions shift in the opposite direction, and displacement vectors from all unit cells were calculated from the standard position to the positive charge center. Polarization, *P*, can be simply calculated by the following equation, $P = \frac{1}{V}\sum_i {\delta _iZ_i}$, where *V* is the unit cell volume, and *δ*_*i*_ and *Z*_*i*_ are the displacement length and effective charge of atom *i*, respectively. Born effective charges for CaTiO_3_ and LaAlO_3_ are adopted to reflect electronic and ionic polarization^48–50^. *V* (= *a*^2^*c*) was calculated from *a* and *c*, which corresponds to the $\sqrt 2$[110] and [001] Ca–Ca (or La–La) spacing of perovskite structure, respectively.

Figure S12 shows the simulated ABF-STEM images of the 10 OOR patterns of CaTiO_3_ considered in Fig. 1c. All ABF-STEM simulations were conducted with the multi-slice method using Dr. Probe software (Ernst Ruska-Centre, Germany)^51^, based on the atomic structures given by our DFT calculations. Figure S13 shows a comprehensive STEM data set of CaTiO_3_/LaAlO_3_ (111) and CaTiO_3_/LaAlO_3_ (001) heterostructures. Discussions on the CaTiO_3_/LaAlO_3_ (111) thin films are provided in the main text. For the CaTiO_3_/LaAlO_3_ (001) heterostructure, the bulk-like nonpolar *Pnma* structure is clear. A high-quality hetero-interfacial structure is shown in Fig. S13i. Figure S13j shows the good agreement between the simulated ABF-STEM of *a*^–^*b*^+^*a*^–^ and the structure of CaTiO_3_. Note that both relaxed CaTiO_3_/LaAlO_3_ (111) (Fig. S13e) and CaTiO_3_/LaAlO_3_ (001) (Fig. S13i) have an *a*^–^*b*^+^*a*^–^ OOR pattern, but they are oriented along the [101] and [$1\bar 10$] directions, respectively. Convolutional neural network (CNN; Fig. S13k) and polarization mapping (Fig. S13l) evidence the *Pnma* structure of CaTiO_3_ grown on an LaAlO_3_ (001) substrate.

*R*3*c*-CaTiO_3_ films can exhibit domains, following the intrinsic twin domains of the underlying LaAlO_3_ (111) single crystal substrate. We experimentally observed all four different domains of *R*3*c*-CaTiO_3_, which is numbered in Fig. S18a. For the domains #1 and #4, we can easily observe the polarization in STEM because the polarization is perpendicular to the [$1\bar 10$] zone axis (Fig. S18b). For the domains #2 and #3, however, polarization vectors have [$1\bar 10$]-components and we barely observe the polarization in STEM (Fig. S18b).

Given the threefold rotational symmetry of LaAlO_3_ (111) substrate, there can be three equivalent domains of the relaxed *Pnma*-CaTiO_3_ on LaAlO_3_ (111). We refer to the three domains as *a*^–^*b*^+^*a*^–^, *a*^+^*b*^–^*b*^–^, and *a*^–^*a*^–^*c*^+^ domains, respectively, as shown in Fig. S19. Since only *a*^–^*b*^+^*a*^–^ shows the characteristic zigzag-like pattern of oxygen, we mainly discussed the *a*^–^*b*^+^*a*^–^ in the main text.

### Deep neural network analysis

The obtained STEM images were filtered to reduce background noise and to extract each atomic column position with sub-pixel accuracy. Denoising autoencoder (DAE), a deep machine learning technique for reconstructing images, was applied to avoid the image distortion caused by a conventional Fourier transformation filter. Each STEM image was sliced into image patches containing a single atom, as training or input data for DAE. Atomic image patches were reconstructed (output) through unsupervised training. The DAE was trained to minimize the difference between the input noisy image and the output reconstructed image. After a sufficient training process, DAE was trained to reconstruct only the atomic image, and not to imitate the noise from the input image. Reconstructed image patches with sharp atomic coordinate information were assembled to construct a full STEM image.

CNN, as a deep learning image recognition technique, was exploited for OOR pattern mapping. General CNNs are mainly composed of convolutional layer (Conv. Layer), max pooling, and fully connected layer (F.C. Layer) (Fig. S20). A Conv. Layer consists of several filters extracting the features from the input image. The filters are matrices and the elements are continuously renewed to learn which feature to extract during the training process. On the contrary, max pooling does not change during training; it compresses the feature images from previous steps. For efficiency, image compression reduces computing power consumption, and for performance, it desensitizes decision making to the small variance of image position. F.C. Layer is composed of several neurons, which make a definitive prediction of OOR, based on the extracted features. Neurons in F.C. Layer are matrices and change via training, like the filters in Conv. Layer.

The main challenge in the application of CNN to STEM imaging is the insufficient quantity of unambiguous training data. For this reason, simulated data were used for the training set. To exclude ambiguity, ABF-STEM simulation was conducted on DFT calculated structures along [$1\bar 10$] projections of the *a*^–^*a*^–^*a*^–^ and *a*^–^*b*^+^*a*^–^ patterns in CaTiO_3_. For the purpose of emulating real experimental conditions and improving the training data, simulated images were preprocessed^52^ (Fig. S21). During the preprocessing steps, a 1 × 2 unit cell, which is the minimum criterion for OOR determination, was cropped to a random size in a random position, and then uniformly resized to 90 × 72 pixels, the input dimension for CNN. Resized images were convolved with probe sizes from 10 to 50 pm, which is the range between the ideal case (10 pm) and the technological limitation (50 pm). Lastly, artificial Gaussian noise was added with random values (between 40% and 60% of the data). These preprocessing steps reproduced 1350 images from a single simulation image.

For OOR identification through the information hidden by nontrivial noise, a custom deep neural network was devised (Fig. S22). This network possessed three Conv. Layers with 32, 64, and 64 filters (5 × 5, 3 × 3 and 3 × 3, respectively) that slide across the whole of each input image. Max pooling layers were connected to the second and the third Conv. Layers. CNN ended up with two F.C. Layers, which had 128 and 2 neurons each. The latter layer implies a binary classification of *a*^–^*a*^–^*a*^–^ or *a*^–^*b*^+^*a*^–^ regardless of the projection of each OOR pattern.

For OOR mapping of the experimental ABF-STEM image, 1 × 2 unit cell images were cropped into image patches that slide across the whole image. Each image patch was resized to 90 × 72, which is the original input size of the custom CNN. Each patch returned its coordinates and OOR mapping result as a probability of each OOR pattern. Based on the coordinates, the patches were reintegrated with the color-mapped prediction result. The deep learning works and the calculations of atomic polarization were all performed based on Python. Also, Keras with TensorFlow backend was utilized as a deep learning library for training the model.

## Supplementary information

Supplementary Information

Peer Review File

## Data Availability

All relevant data presented in this paper are available from the authors upon reasonable request. Source data are provided with this paper.

## Source data

Source Data
