# Supplementary material for: Stabilizing hidden room-temperature ferroelectricity via a metastable atomic distortion pattern
Source: Nat Commun. 2020 Oct 2;11:4944. doi: 10.1038/s41467-020-18741-w (PMC7532175; doi:10.1038/s41467-020-18741-w)
Supplement: Supplementary file 1 — Supplementary Information [file 41467_2020_18741_MOESM1_ESM.pdf]

Supplementary Information

“Stabilizing hidden room-temperature ferroelectricity via a metastable atomic distortion pattern”

Jeong Rae Kim et al.

Supplementary Figures

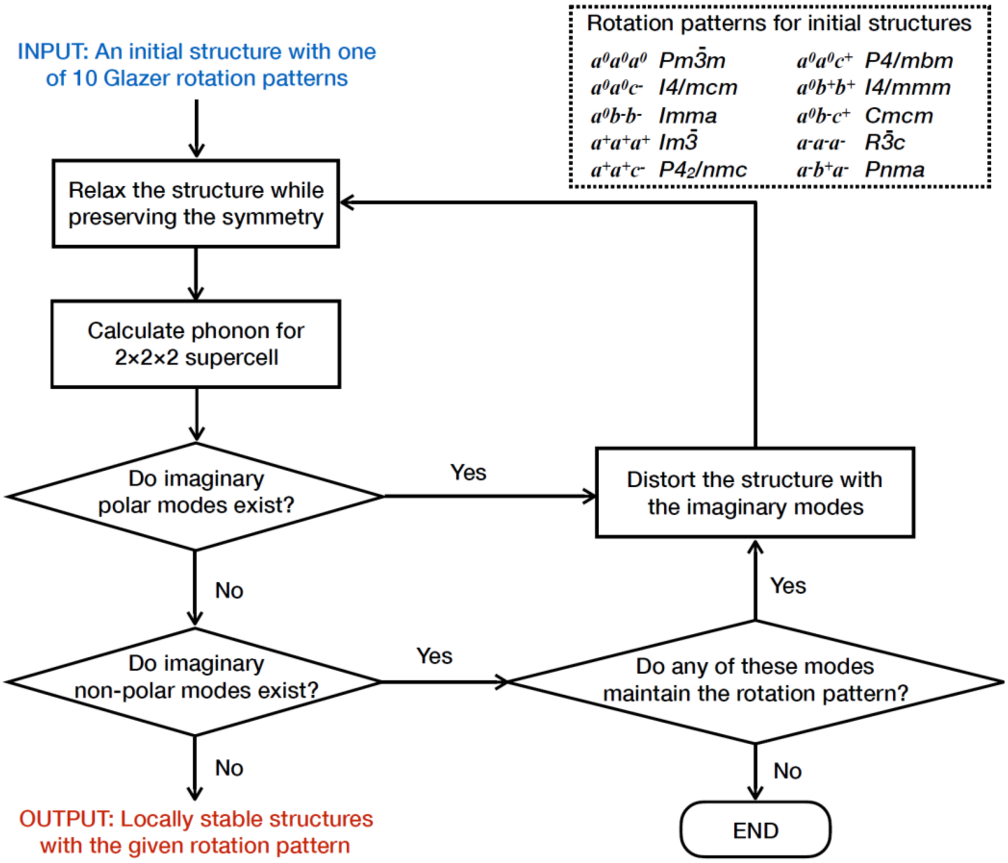

Fig. S1 | Flowchart of the method used to obtain locally stable polar structures of $CaTiO_3$. Procedure to obtain locally-stable polar structures (*R*3*c*, *P*4_2*/mc*, *P*4*mm*, *Amm*2, and *R*3*m*). For this, we considered 10 OOR patterns, as shown in Fig. 1c.

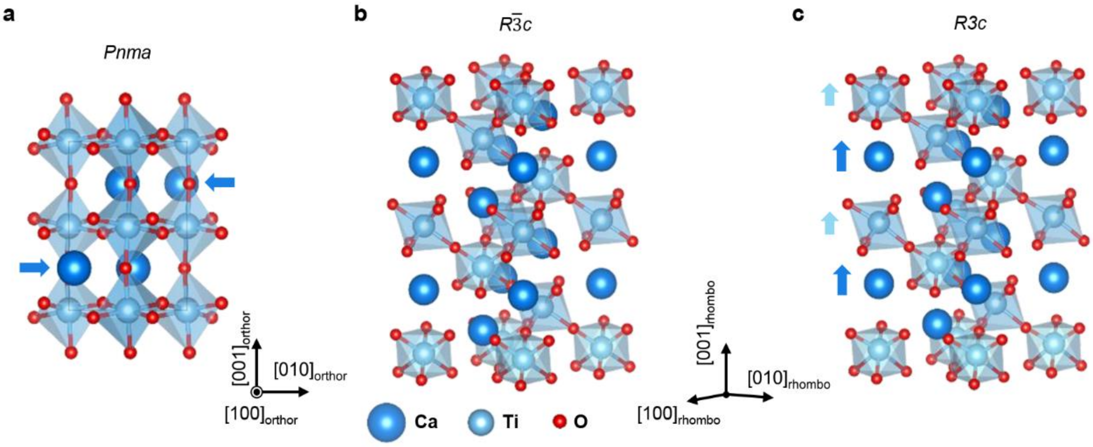

Fig. S2 | DFT calculation of structures of $CaTiO_3$. a,b,c, DFT calculation of $CaTiO_3$ with *Pnma* **(a)**, $R\bar{3}c$ **(b)**, and *R*3*c* **(c)** structures.

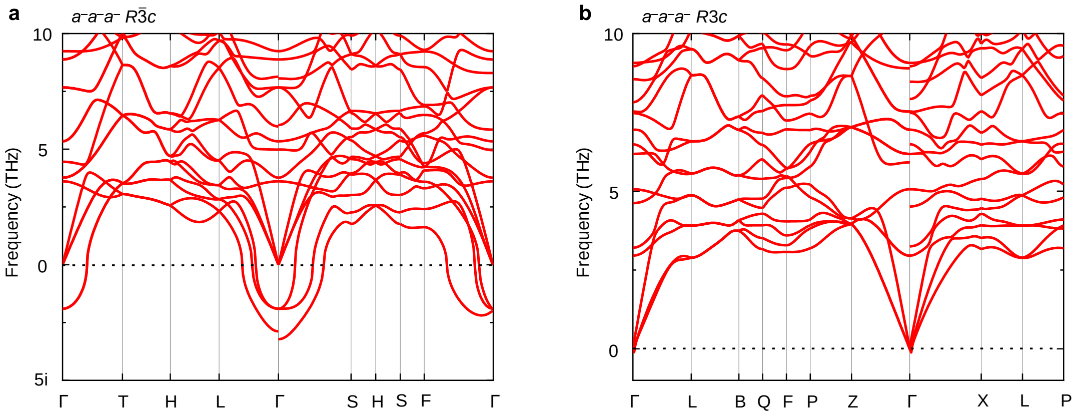

Fig. S3 | Phonon dispersions of $CaTiO_3$ with $a^-a^-a^-$ OOR pattern. a,**b**, Phonon dispersion of $CaTiO_3$ with $R\bar{3}c$ (**a**) and $R3c$ (**b**). The presence of imaginary phonons in the $R\bar{3}c$ structure indicates structural instability. The absence of an imaginary phonon in the $R3c$ structure shows the local stability of the structure.

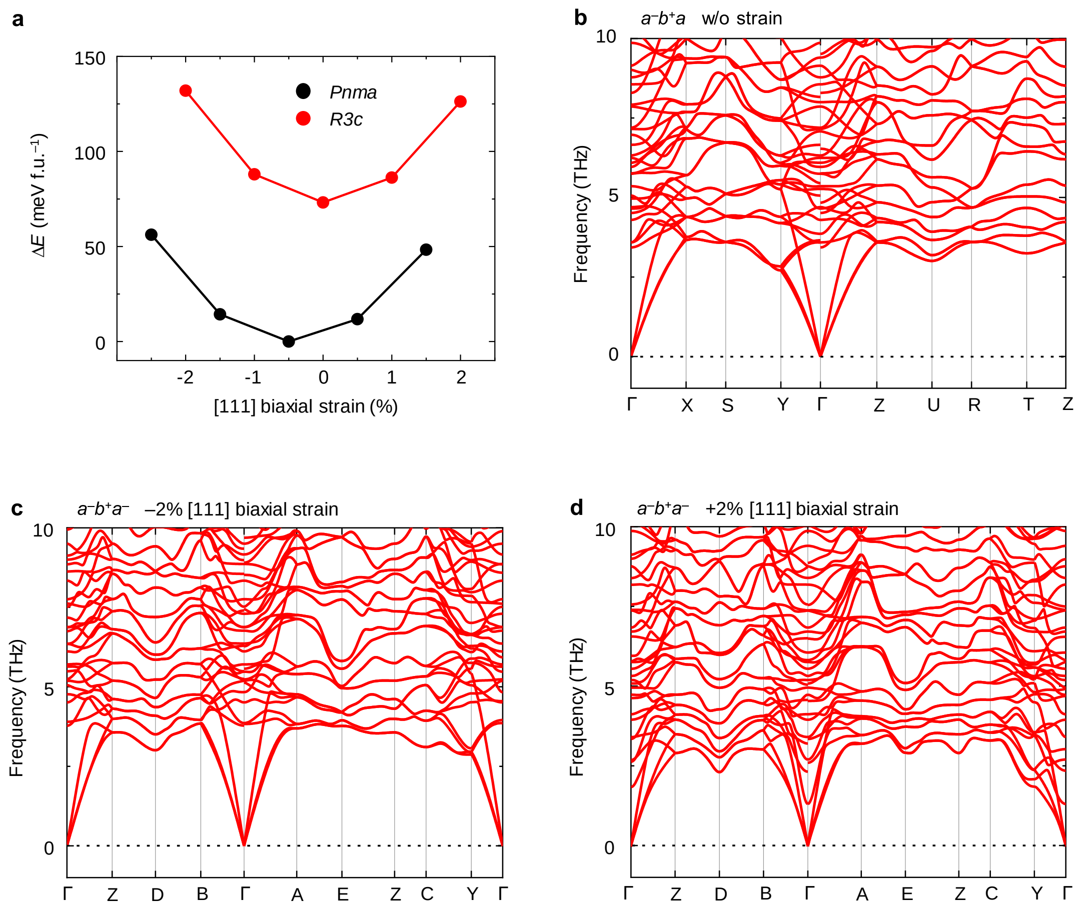

Fig. S4 | Stability of *Pnma*-$CaTiO_3$ under [111]-oriented biaxial strain. a, Energy of *Pnma*-$CaTiO_3$ and *R*3*c*–$CaTiO_3$ under [111]-oriented biaxial strain. *Pnma*-$CaTiO_3$ always has lower energy than *R*3*c*-$CaTiO_3$. **b–d,** Phonon dispersions of *Pnma*-$CaTiO_3$ under [111]-oriented biaxial strains of 0% (**b**), –2% (**c**), and +2% (**d**). No imaginary phonon is observed, implying that simple strain engineering can neither change the OOR pattern of $CaTiO_3$ nor induce ferroelectricity.

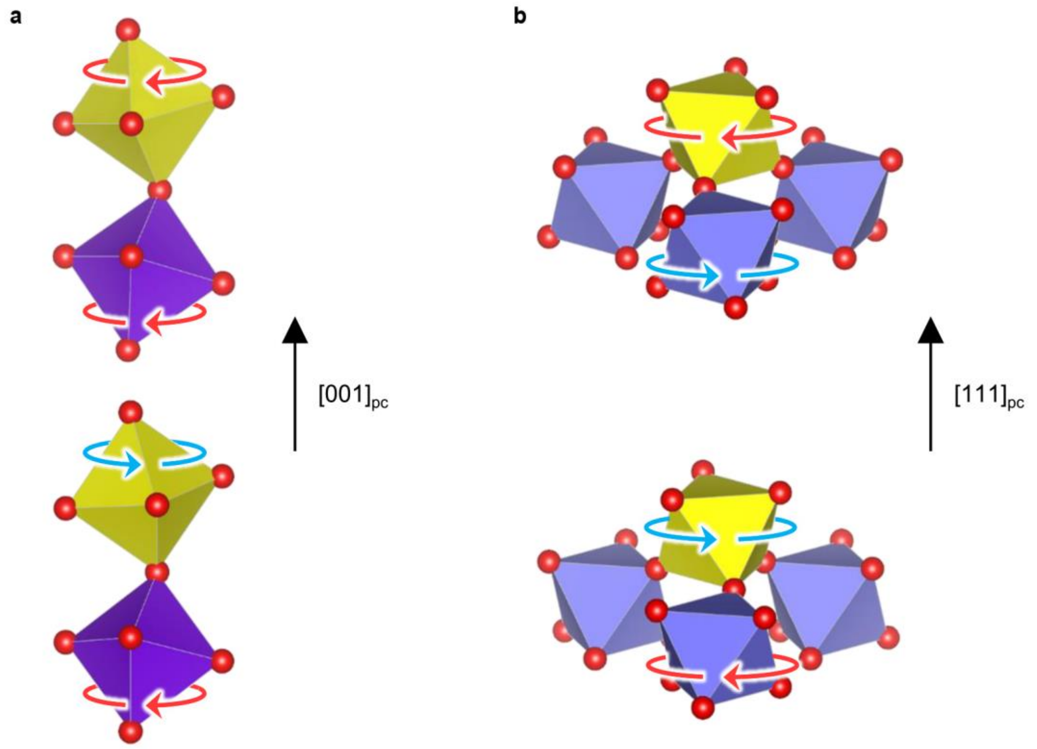

Fig. S5 | Octahedral connectivity across (001)$_{pc}$ and (111)$_{pc}$ heterointerface. a, Octahedral connectivity across (001)$_{pc}$–oriented heterointerface. One bond connection allows the interfacial OOR relationships to be either in-phase or out-of-phase. **b**, Octahedral connectivity across (111)$_{pc}$–oriented heterointerface. Three bond connections lock the interfacial OOR pattern configurations.

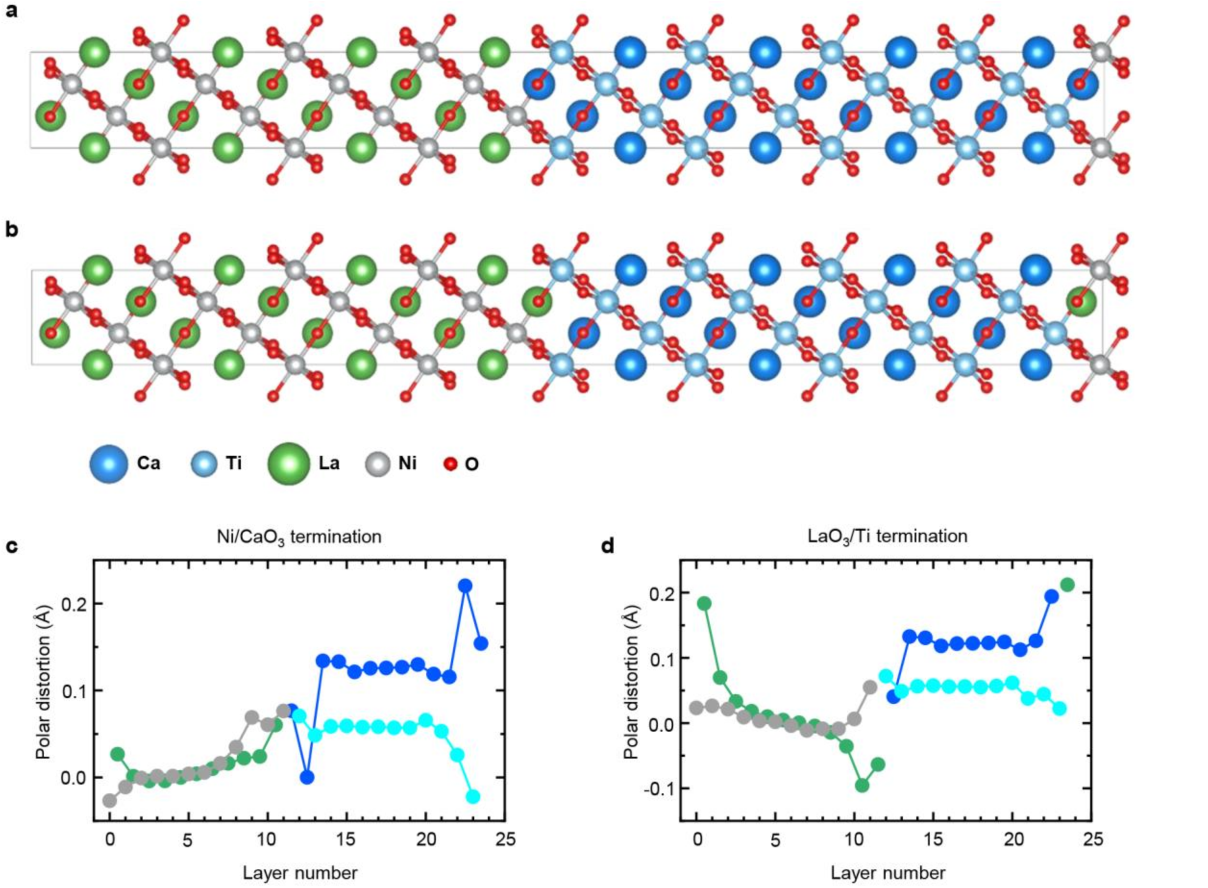

Fig. S6 | DFT calculation of interface electric dipole at $LaNiO_3$/$CaTiO_3$ interface. a,b, Schematic of [$(LaNiO_3)_{12}$/$(CaTiO_3)_{12}$] superlattices for DFT calculation with Ni/CaO_3 **(a)** and LaO_3/Ti **(b)** interface terminations, respectively. **c,d,** DFT calculation of layer-resolved ionic displacement in the Ni/CaO_3–terminated **(c)** and LaO_3/Ti–terminated **(d)** [$(LaNiO_3)_{12}$/$(CaTiO_3)_{12}$] superlattices.

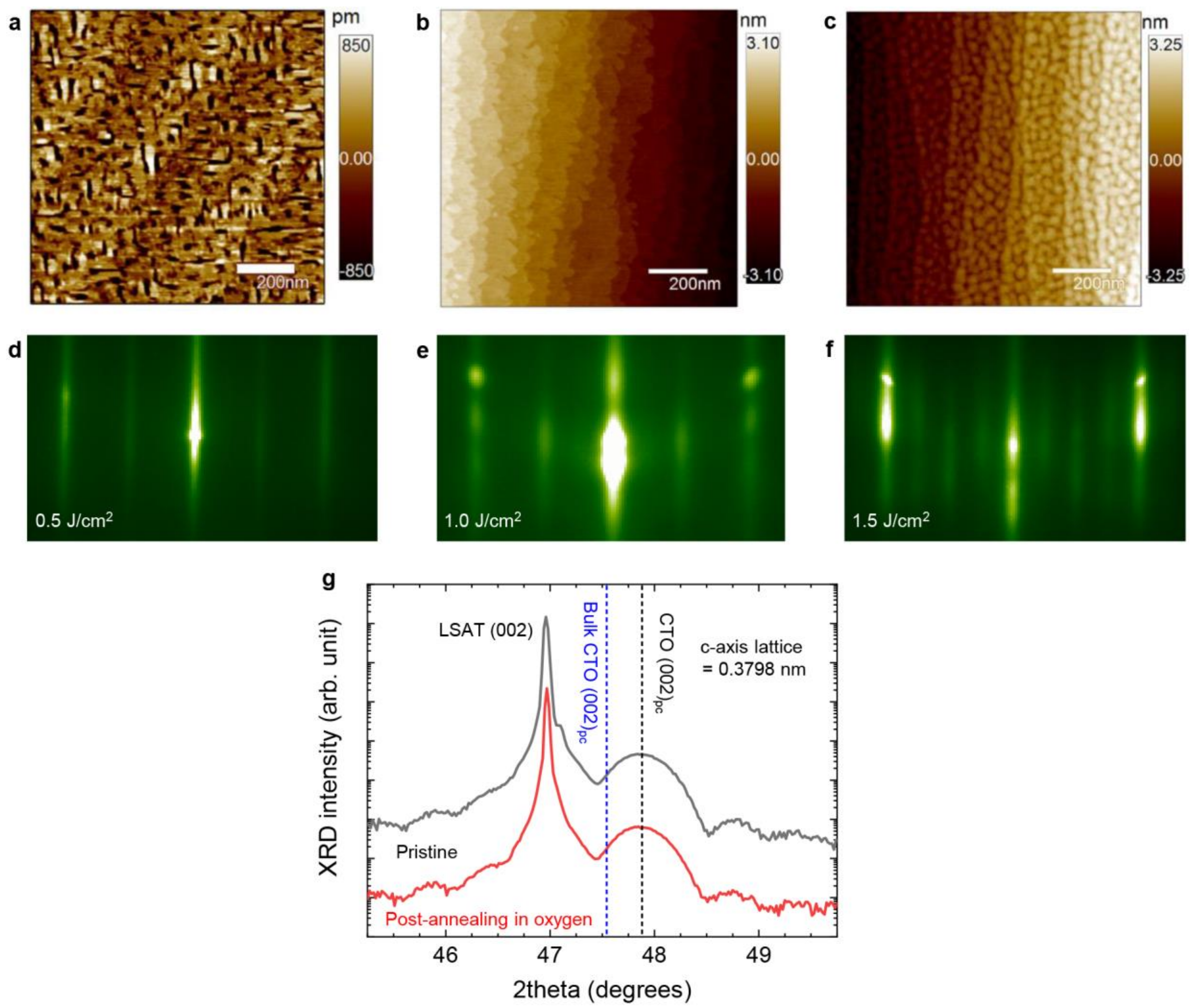

Fig. S7 | Optimization of cation stoichiometry of $CaTiO_3$ thin films using pulsed laser deposition (PLD). a–c, Atomic force microscopy (AFM) height images of 20 nm-thick $CaTiO_3$/$LaAlO_3$ (001) thin films grown by PLD with laser energy density of 0.5 (**a**), 1.0 (**b**), and 1.5 J cm^{-2} (**c**). **d–f**, Reflection high energy electron diffraction (RHEED) pattern images of films grown with laser energy density of 0.5 (**d**), 1.0 (**e**), and 1.5 J cm^{-2} (**f**). **g**, X-ray theta-2theta scan of 20 nm-thick $CaTiO_3$ thin film grown on a $(LaAlO_3)_{0.3}$-$(Sr_2AlTaO_6)_{0.7}$ (LSAT) (001) substrate with a laser energy density of 1.0 J cm^{-2}. The black and red curves correspond to the pristine $CaTiO_3$ film and the post-annealed (in an oxygen environment) $CaTiO_3$ film, respectively.

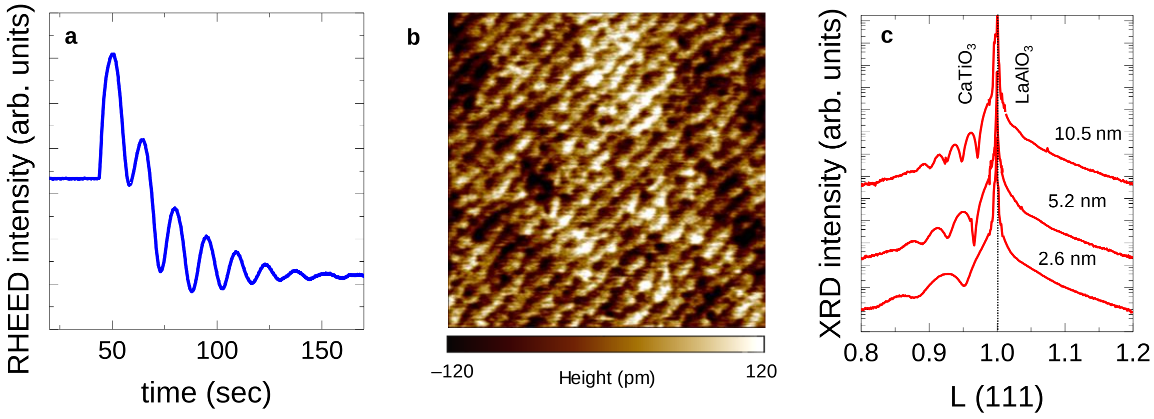

Fig. S8 | Characterization of $CaTiO_3$/$LaAlO_3$ (111) thin films. a, RHEED intensity monitoring showing layer-by-layer growth of $CaTiO_3$ thin film on $LaAlO_3$ (111) substrate. **b**, AFM height image of 2.5 nm-thick $CaTiO_3$/$LaAlO_3$ (111) thin film. A smooth surface with step-terrace structures is present. **c**, X-ray diffraction (XRD) data on $CaTiO_3$/$LaAlO_3$ (111) thin films, measured around $LaAlO_3$ (111) diffraction peaks. $CaTiO_3$ are epitaxially grown on $LaAlO_3$ (111) substrates.

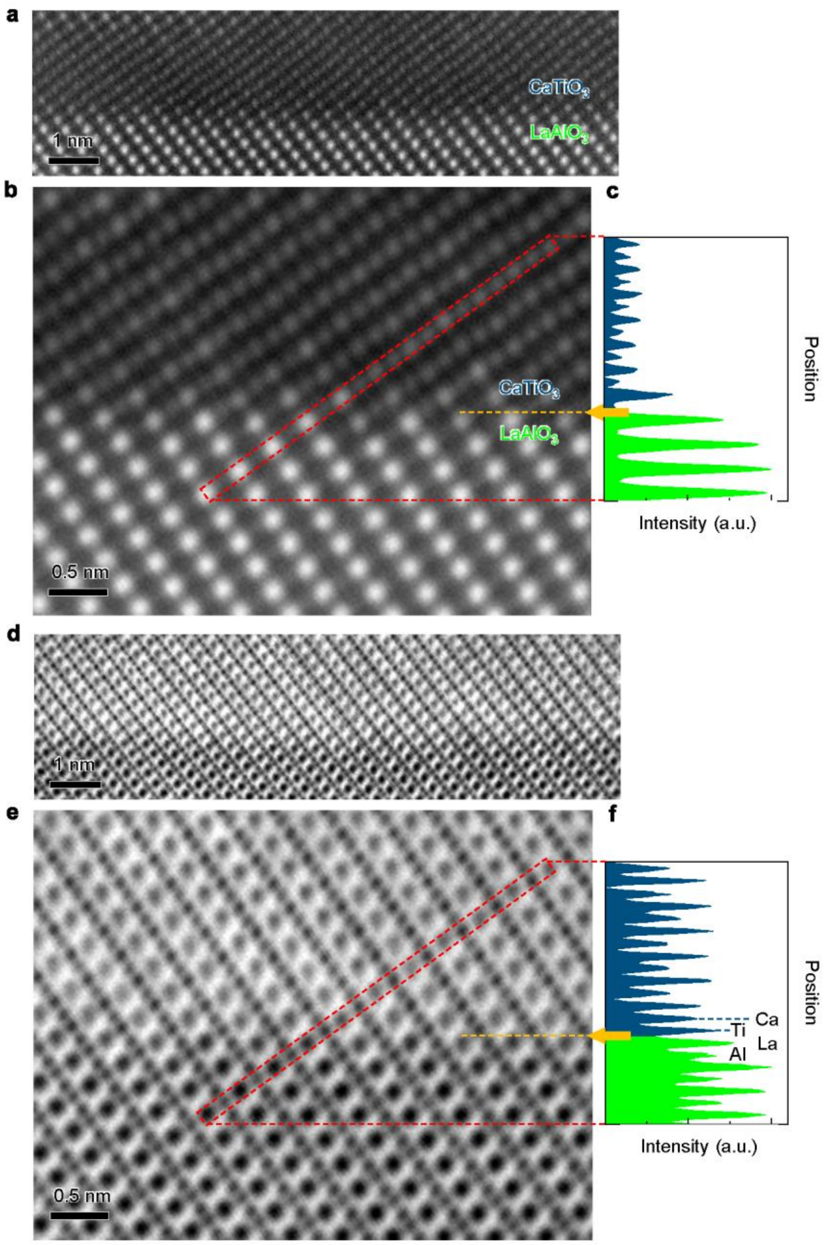

Fig. S9 | Abrupt heterointerface between $CaTiO_3$ and $LaAlO_3$ in $CaTiO_3/LaAlO_3$ (111) heterostructure. **a**,**d**, High angle annular dark field- (HAADF) and annular bright-field scanning transmission electron microscopy (ABF-STEM) images of $CaTiO_3/LaAlO_3$ (111) heterostructure along zone axis of $[1\bar{1}0]$. In the observed region, no notable defects or structural distortions are seen. **b**,**e**, HAADF- and ABF-STEM images of $CaTiO_3/LaAlO_3$ (111) heterostructure with different length scale. **c**,**f**, Line profiles of HAADF- (**c**) and ABF-STEM (**f**) intensity along the red dashed boxes in **b** and **e**.

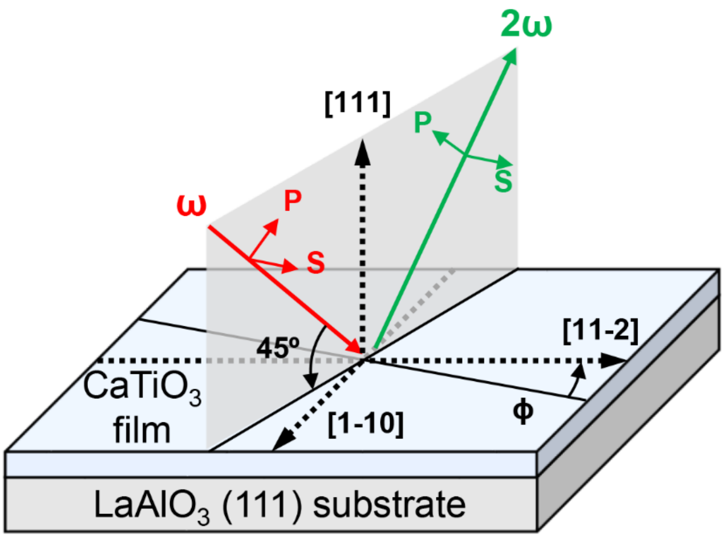

Fig. S10 | Experimental set-up for reflection second harmonic generation. Reflection second harmonic generation where the 800-nm femtosecond wave is irradiated on the $CaTiO_3$ thin films with an incidence angle of 45 degrees. "p" ("s") indicates parallel (perpendicular) polarization with respect to the plane of incidence.

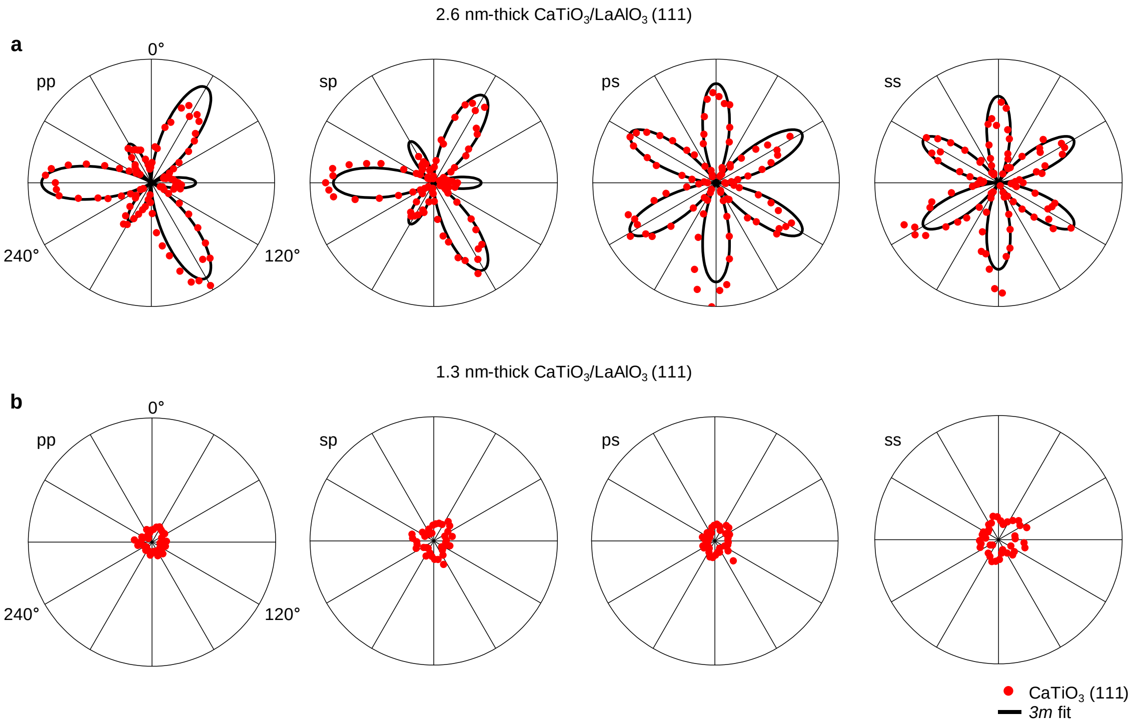

Fig. S11 | Critical thickness for ferroelectricity in $CaTiO_3$/$LaAlO_3$ (111) thin films. a,b, Optical second harmonic generation (SHG) signals from 2.6 nm- (**a**) and 1.3 nm-thick (**b**) $CaTiO_3$/$LaAlO_3$ (111) thin films. "p" ("s") indicates parallel (perpendicular) polarization with respect to the plane of incidence.

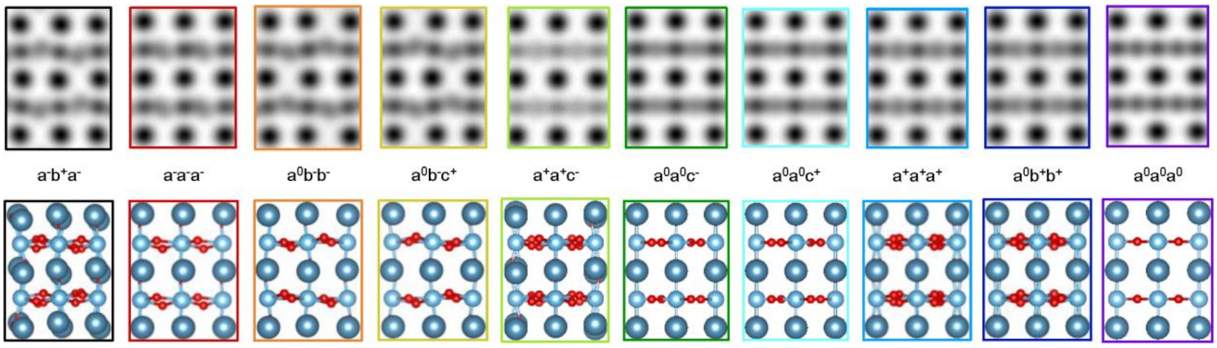

Fig. S12 | Simulated ABF-STEM images of $CaTiO_3$ for various OOR patterns. Simulated ABF-STEM images (multi-slice simulation performed using Dr. Probe software) and corresponding atomic structures of $CaTiO_3$ along zone axis $[1\bar{1}0]$ for various OOR patterns. Due to BO_6 rotation and differences in oxygen column density, the images no longer have a round-shaped oxygen column, except for $a^0a^0a^0$. The target OOR pattern, $a^-a^-a^-$, has the unique feature of diagonally elongated oxygen peaks, lined up horizontally and vertically. Also, a control group ($a^-b^+a^-$) can be distinguished by the tails of its oxygen column and buckling.

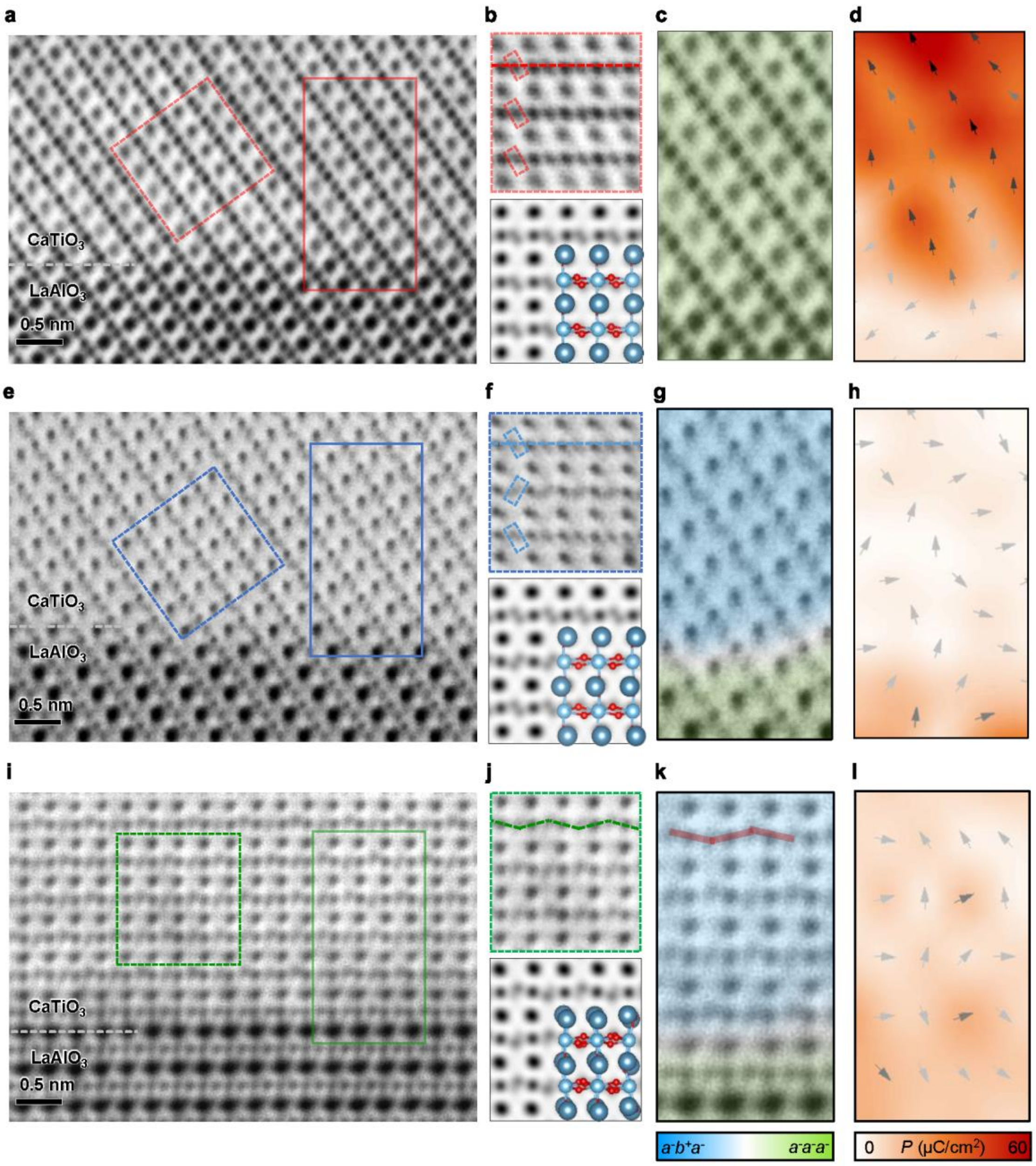

Fig. S13 | Atomic-scale analysis of the OOR pattern and polarity of the whole $CaTiO_3/LaAlO_3$ heterostructure series. a,**e**, ABF-STEM image of $CaTiO_3/LaAlO_3$ (111) heterostructure along zone axis $[1\bar{1}0]$ under electron acceleration voltages of 80 kV (**a**) and 200 kV (**e**). **b**, Magnified and rotated image of the red dashed box (left) in **a**, with the simulated ABF-STEM image of the $a^-a^-a^-$ OOR pattern shown below. **c**, A magnified image of the red solid box (right) in **a** and corresponding OOR pattern of each 1 × 2 unit cell, obtained using a convolutional neural network (CNN). Color indicates the probability (similarity) of each OOR

pattern. **d**, Polarization map of each unit cell in the same region as **c**. Arrows denote the polarization direction and intensity; the stronger the polarization, the darker the arrow. Intensity of polarization is also expressed as a color map, ranging from white (week) to red (strong). **f**, Magnified and rotated image of the blue dashed box (left) in **e**, with the simulated ABF-STEM image of $a^{-}b^{+}a^{-}$ OOR pattern shown below. **g**, Magnified image of the blue solid box (right) in **e** and corresponding OOR map of the CNN. **h**, Polarization map of the same region shown in **g**. **i**, ABF-STEM image of $CaTiO_3/LaAlO_3$ (001) heterostructure along zone axis $[1\bar{1}0]$ under an electron acceleration voltage of 200 kV. **j**, A magnified image of the green dashed box (left) in **i**, with the simulated ABF-STEM image of the $a^{-}b^{+}a^{-}$ OOR pattern shown below. **k**, Magnified image of the green solid box (right) in **i** and corresponding OOR map of the CNN. **l**, Polarization map of the same region shown in **k.**

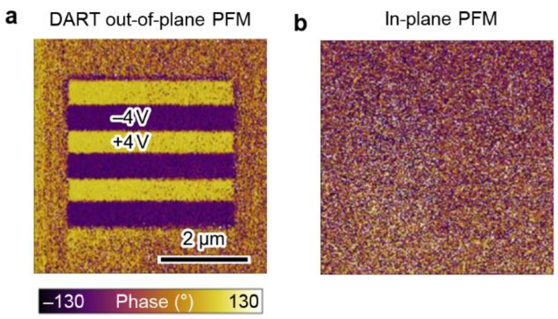

Fig. S14 | In-plane PFM imaging of *R*3*c*-$CaTiO_3$. a, Bipolar, out-of-plane domain pattern of *R*3*c*-$CaTiO_3$ imaged by Dual AC resonance tracking (DART) PFM. **b**, Simultaneously-imaged in-plane domain pattern of *R*3*c*-$CaTiO_3$ by vector PFM mode.

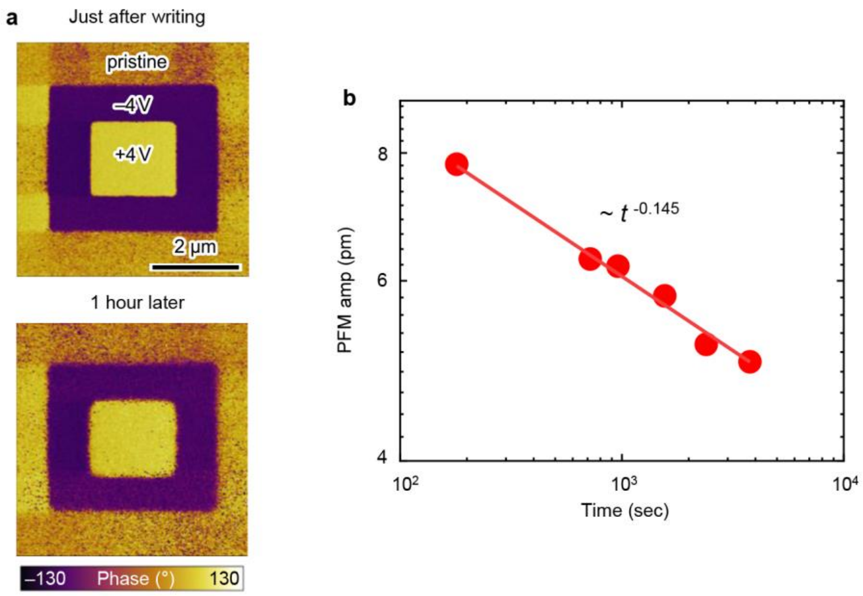

Fig. S15 | Stability of ferroelectric domain of *R*3*c*-CaTiO$_3$. a, Bipolar domain of *R*3*c*-CaTiO$_3$ which persists for an hour. **b**, Time-dependent PFM amplitude of *R*3*c*-CaTiO$_3$ which follow a power-law with an exponent of 0.145.

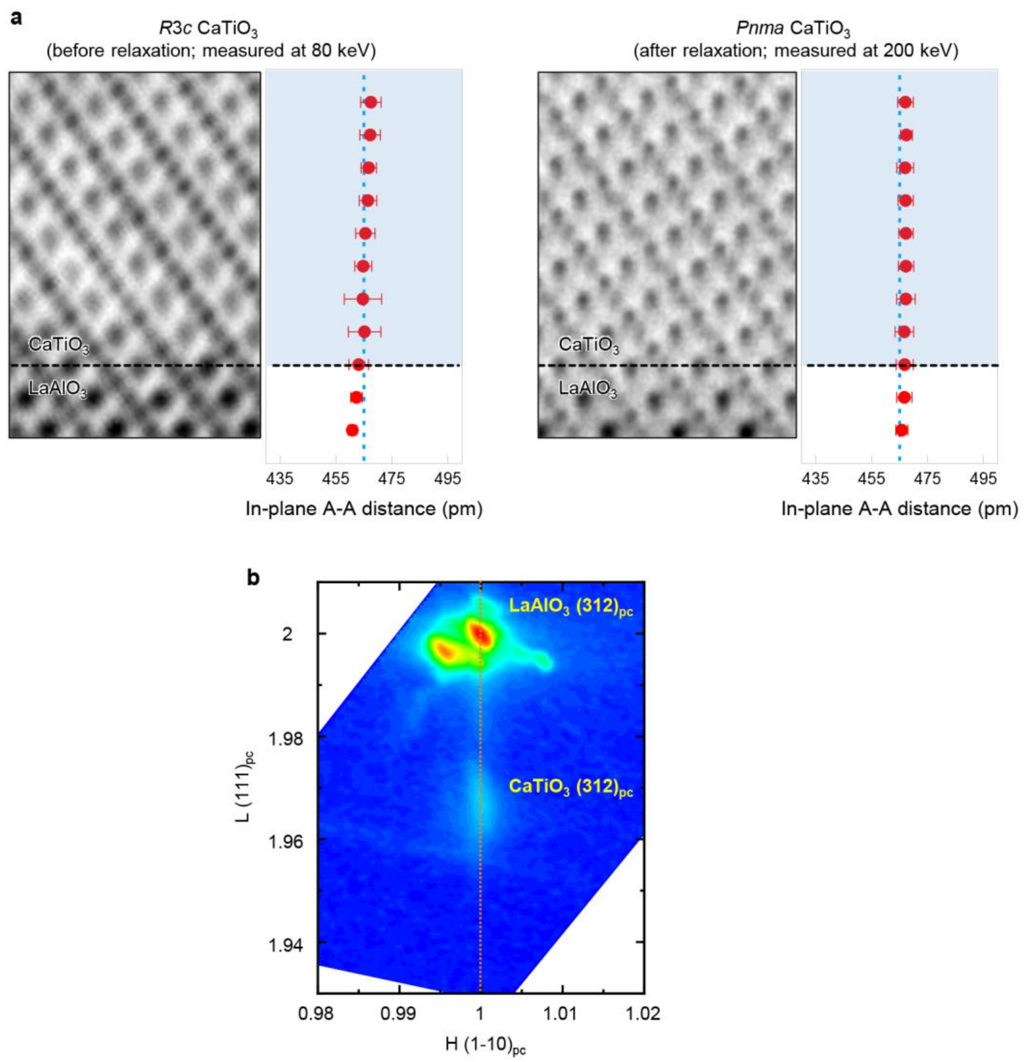

Fig. S16 | Fully-strained $CaTiO_3$/$LaAlO_3$ (111)$_{pc}$ thin film. a, $CaTiO_3$ in-plane lattice before and after the OOR-pattern relaxation. **b**, Reciprocal space mapping of 25nm-thick $CaTiO_3$/$LaAlO_3$ (111)$_{pc}$ thin film near $LaAlO_3$ (312)$_{pc}$ peak. $CaTiO_3$ (312)$_{pc}$ and $LaAlO_3$ (312)$_{pc}$ are peaked at an identical H value, meaning identical in-plane lattice parameters.

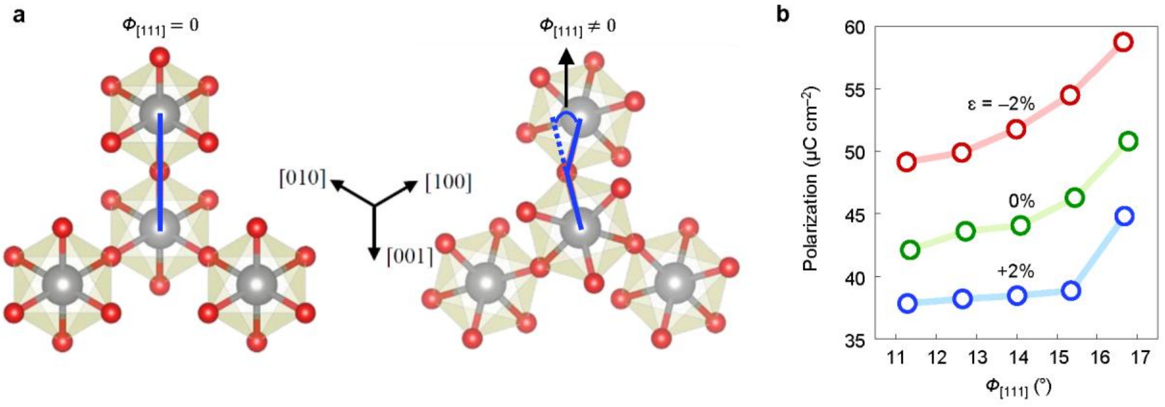

Fig. S17 | Cooperative coupling between ferroelectricity and OOR in *R*3*c*-$CaTiO_3$. a, Definition of OOR angle $\Phi_{[111]}$ of *R*3*c*-$CaTiO_3$. **b,** $\Phi_{[111]}$- and strain-dependent ferroelectric polarization in *R*3*c*-$CaTiO_3$. The polarization value increases with the OOR angle, showing cooperative coupling between ferroelectricity and OOR.

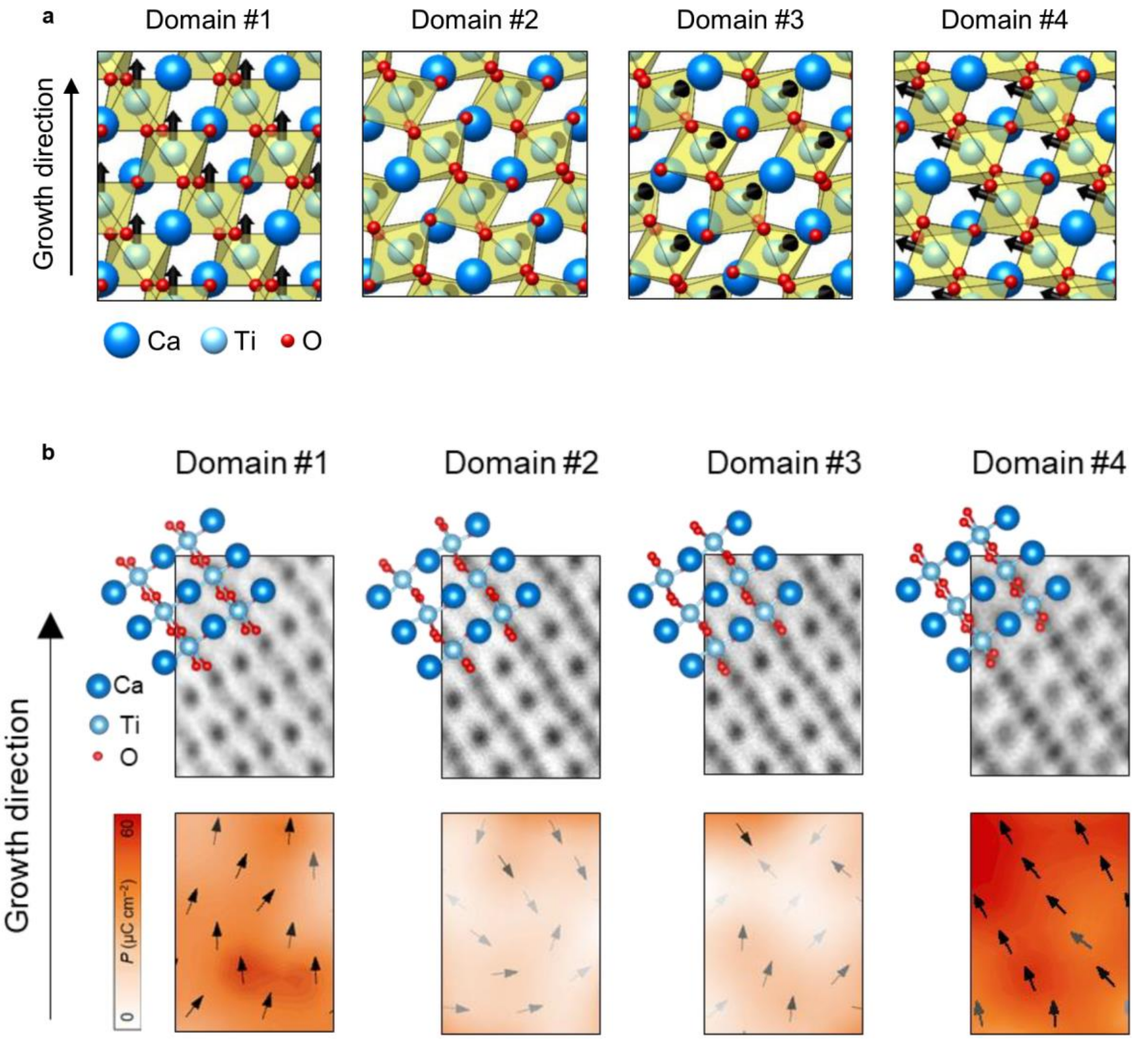

Fig. S18 | Domain structures of *R*3*c*-$CaTiO_3$ stabilized on $LaAlO_3$ $(111)_{pc}$ substrate. a, Schematic of four possible domains of *R*3*c*-$CaTiO_3$. Black arrows indicate the Ti polar displacement. **b,** Atomic scale imaging and polarization mapping on four domains of *R*3*c*-$CaTiO_3$ via ABF-STEM.

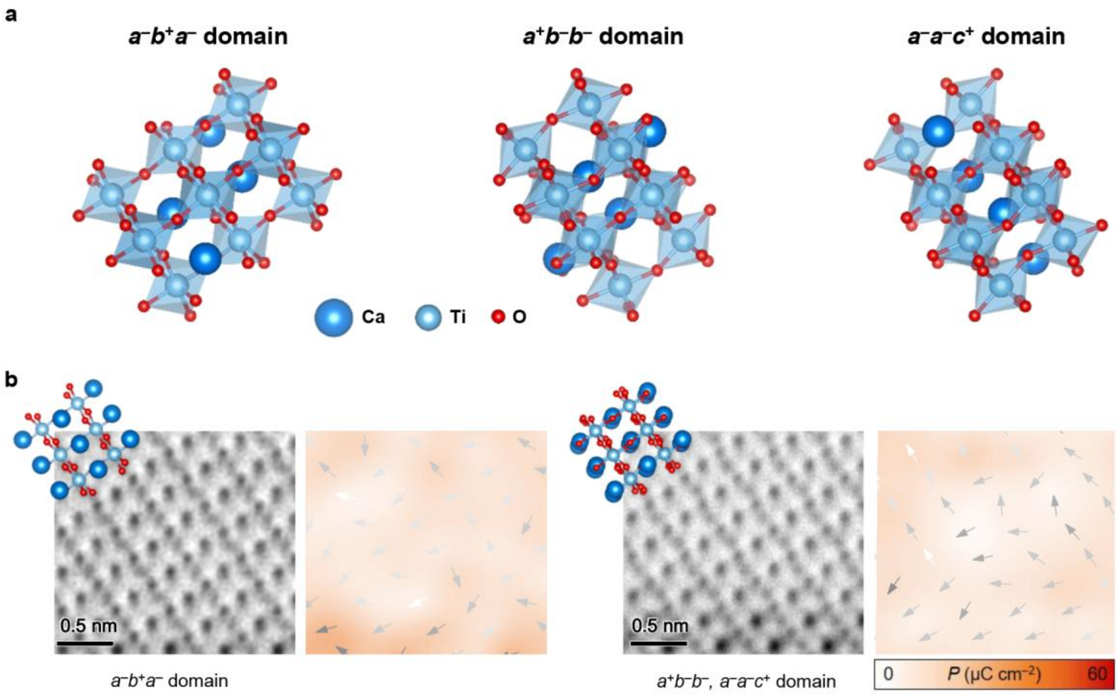

Fig. S19 | Domain structures of *Pnma*-$CaTiO_3$ on $LaAlO_3$ $(111)_{pc}$ substrate. a, Schematic of three possible domain structures of *Pnma*-$CaTiO_3$ thin films on $(111)_{pc}$-plane of $LaAlO_3$. **b,** Atomic scale imaging and polarization mapping on three domain structures of *Pnma*-$CaTiO_3$ via ABF-STEM.

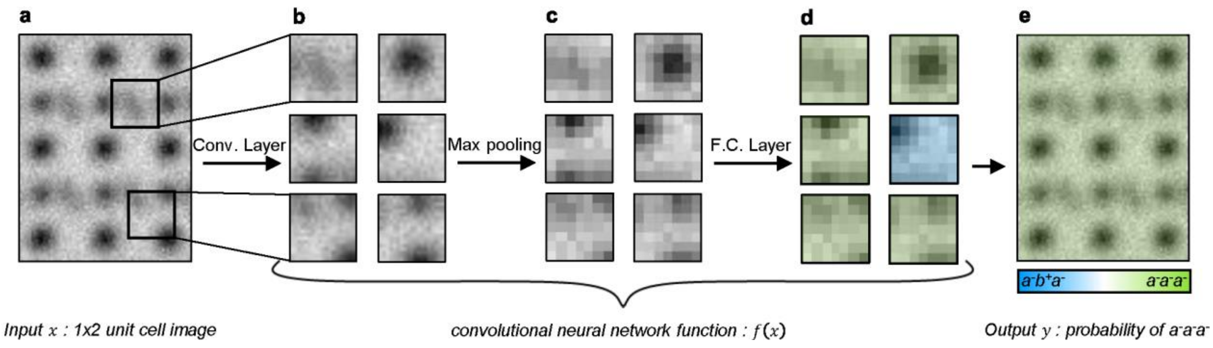

Fig. S20 | Characteristics of the convolutional neural network (CNN). a, An example ABF-STEM simulation in the $[1\bar{1}0]$ projections of $a^-a^-a^-$ for training the CNN. **b**, Feature images extracted by filters in Convolutional layer (Conv. Layer). **c**, Feature images compresed by max pooling. **d**, Estimation of the similarity of each feature to each OOR in a fully connected layer (F.C. layer). **b**–**d** are exaggerated for explanation and the feature images are geometrical, which humans cannot distinguish. **e**, Plot of OOR mapping in the 1×2 unit cell in the $[1\bar{1}0]$ projection. Color indicates the probability (similarity) of each OOR pattern, as calculated by the CNN.

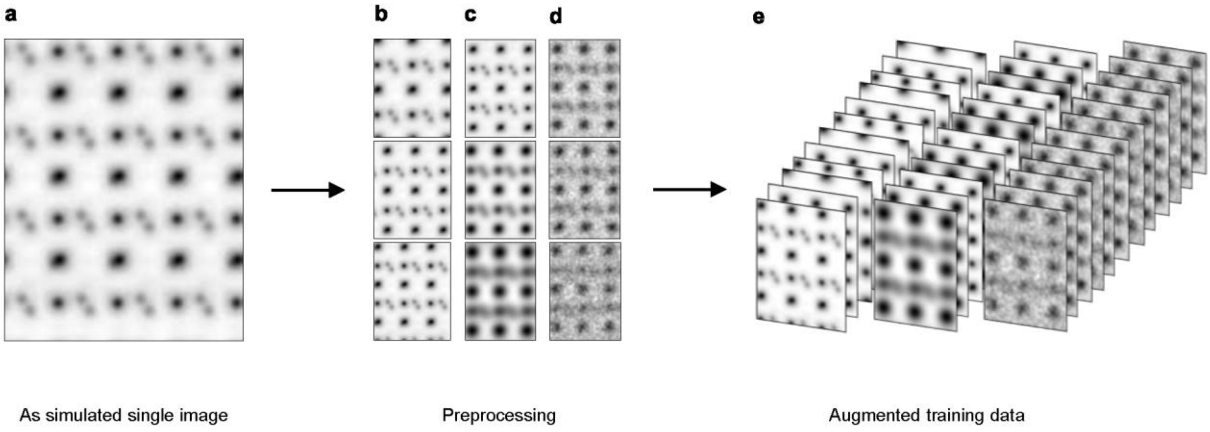

Fig. S21 | Training data augmentation. The main challenge when applying CNNs to STEM imaging is the lack of a sufficient amount of training data with clear data labels. To avoid ambiguity during the training of our CNN, ABF-STEM simulations with density functional theory (DFT)-calculated structures, each with an unambiguous data label, were used as the training data. An ABF-STEM simulation in the $[1\bar{1}0]$ projection of $a^-b^+a^-$ and $a^-a^-a^-$ OORs of $CaTiO_3$ was conducted with a multi-slice technique; **a** shows an example $[1\bar{1}0]$ projection image of $a^-a^-a^-$. To acquire a vast amount of training data and emulate experimental conditions from an ideal simulated image, the original image undergoes pre-processing steps, shown in **b**–**d**. **b**, 1 × 2 unit cell of a perovskite structure, which is the minimum criterion for OOR determination, was cropped in a random position, to a random size. The cropped images were uniformly resized to 90 × 72, which is equal to the input dimension for the CNN. **c**, The beam probe size of STEM was varied from 10 to 50 pm, which presents the range between the ideal case (10 pm) and the technological limitation (50 pm). **d**, Artificial Gaussian noise was added to emulate an experimental STEM image. The proportion of noise was randomly varied between 40% and 60% for all of the training data. **e**, An example of augmented data from a single simulated ABF-STEM image shown in **a**. Through pre-processing steps, one projection of an OOR pattern is augmented to yield 1,350 images.

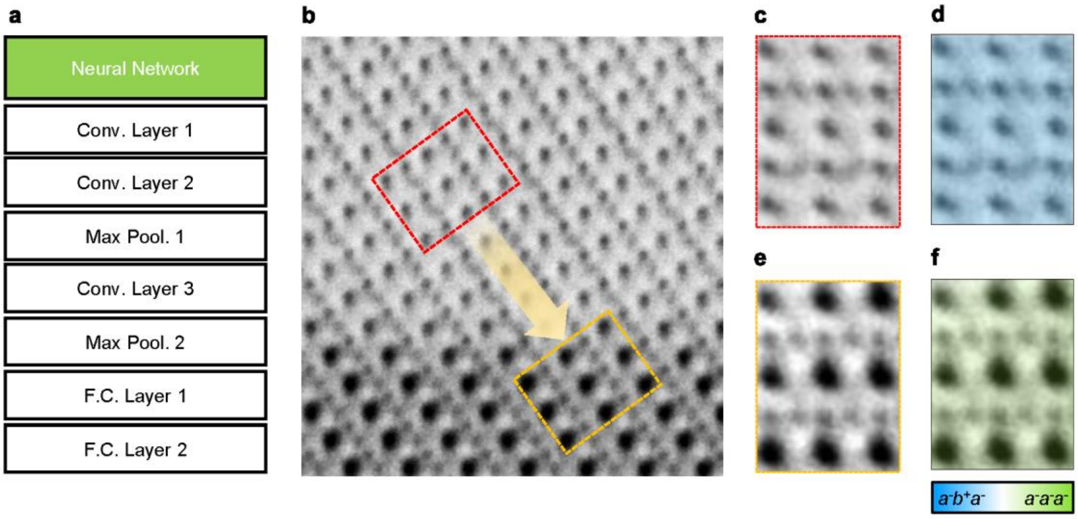

Fig. S22 | Workflow of OOR mapping and the architecture of the CNN. a, The architecture of the custom CNN used in this study. The layers, differing in type and dimensions, were connected from top to bottom. **b**, An example experimental ABF-STEM image used as input for the OOR mapping. **c**,**e**, The 1 × 2 unit cells are cropped from the red and yellow dashed boxes in **b**. For OOR mapping, each 1 × 2 unit cell is cropped from **b**. **d**,**f**, An OOR map is plotted on inputs **c** and **e**. Color indicates the probability (similarity) of each OOR pattern.

Supplementary Table

Table S1 | Table of lattice parameters and strain values from DFT

Type	Hexagonal		Orthorhombic			$(Vol./f.u.)^{1/3}$
	a	c	a	b	c	a
$LaAlO_3$	5.315	12.994	N/A	N/A	N/A	3.756
$Pnma$-$CaTiO_3$	N/A	N/A	5.397	7.527	5.288	3.773
Strain (%)	N/A	N/A	N/A	N/A	N/A	-0.45
$R\bar{3}c$-$CaTiO_3$	5.370	12.990	N/A	N/A	N/A	3.781
Strain (%)	-1.02	0.03	N/A	N/A	N/A	-0.67
$R3c$-$CaTiO_3$	5.360	13.133	N/A	N/A	N/A	3.791
Strain (%)	-0.84	-1.06	N/A	N/A	N/A	-0.91
